# High-Quality Genomes and High-Density Genetic Map Facilitate the Identification of Genes From a Weedy Rice

**DOI:** 10.3389/fpls.2021.775051

**Published:** 2021-11-19

**Authors:** Fei Li, Zhenyun Han, Weihua Qiao, Junrui Wang, Yue Song, Yongxia Cui, Jiaqi Li, Jinyue Ge, Danjing Lou, Weiya Fan, Danting Li, Baoxuan Nong, Zongqiong Zhang, Yunlian Cheng, Lifang Zhang, Xiaoming Zheng, Qingwen Yang

**Affiliations:** ^1^National Key Facility for Crop Gene Resources and Genetic Improvement, Institute of Crop Sciences, Chinese Academy of Agricultural Sciences, Beijing, China; ^2^Guangxi Key Laboratory for Polysaccharide Materials and Modifications, School of Marine Sciences and Biotechnology, Guangxi University for Nationalities, Nanning, China; ^3^School of Clinical Medicine, Southwest Medical University, Luzhou, China; ^4^Little Berry Research Room, Liaoning Institute of Fruit Science, Yingkou, China; ^5^Guangxi Key Laboratory of Rice Genetics and Breeding, Rice Research Institute, Guangxi Academy of Agricultural Sciences, Nanning, China

**Keywords:** weedy rice, genetic map, QTL mapping, reference genome, comparative genomics

## Abstract

Genes have been lost or weakened from cultivated rice during rice domestication and breeding. Weedy rice (*Oryza sativa* f. *spontanea*) is usually recognized as the progeny between cultivated rice and wild rice and is also known to harbor an gene pool for rice breeding. Therefore, identifying genes from weedy rice germplasms is an important way to break the bottleneck of rice breeding. To discover genes from weedy rice germplasms, we constructed a genetic map based on w-hole-genome sequencing of a F_2_ population derived from the cross between LM8 and a cultivated rice variety. We further identified 31 QTLs associated with 12 important agronomic traits and revealed that *ORUFILM03g000095* gene may play an important role in grain length regulation and participate in grain formation. To clarify the genomic characteristics from weedy rice germplasms of LM8, we generated a high-quality genome assembly using single-molecule sequencing, Bionano optical mapping, and Hi-C technologies. The genome harbored a total size of 375.8 Mb, a scaffold N50 of 24.1 Mb, and originated approximately 0.32 million years ago (Mya) and was more closely related to *Oryza sativa* ssp. *japonica*. and contained 672 unique genes. It is related to the formation of grain shape, heading date and tillering. This study generated a high-quality reference genome of weedy rice and high-density genetic map that would benefit the analysis of genome evolution for related species and suggested an effective way to identify genes related to important agronomic traits for further rice breeding.

## Introduction

Cultivated rice is one of the most important staple crops worldwide. The breeding of rice varieties with improved yield, quality, resistance to diseases and pests, and tolerance to abiotic stresses is significant to meet the increasing food demand in China and the world (Khush, 2001; Yang and Hwa, 2008; Xu et al., 2021). However, many genes have been lost from cultivated rice due to the long-term domestication and artificial selection, which hinders the breeding of advanced rice varieties. To the contrary, wild rice growing in natural environments is resistant or tolerant to different biotic and abiotic stresses and therefore retains a natural gene pool containing a large number of genes that have been lost or weakened from cultivated rice (Sun et al., 2002). Weedy rice has many characteristic traits similar to those of wild rice, many studies indicated that weedy rice was originated from wild rice and serves as a transition type between wild rice and cultivated rice (Baker, 1974; Wet and Harlan, 1975; Cho et al., 1995). Previous studies showed that weedy rice harbors the AA genome and no reproductive isolation was observed between weedy rice and cultivated rice (Nadir et al., 2018; Sun et al., 2019). Generally, the genes of weedy rice can be transferred to cultivated rice through breeding techniques such as hybridization and backcrossing (Lu et al., 2000; Stein et al., 2018). Weedy rice has been usually used as the genetic materials for rice genetics and breeding or to identify genes related to stress tolerance, disease and pest resistance, high yield, and high grain quality for improving modern rice varieties (Ishikawa et al., 2005; Shivrain et al., 2010; Dai et al., 2013).

In the past decades, rice functional genomics research, which focuses on technology platform construction and molecular cloning and functional analysis of genes related to important agronomic traits, has resulted in numerous achievements in gene discovery (Han et al., 2007; Xu et al., 2021). Due to its small genome and relatively simple structure, *Oryza sativa* (9311 and Nipponbare) became the first sequenced rice species in 2002 (Goff et al., 2002; Yu et al., 2002). These rice reference genomes have enabled massive rice functional genomics research, accelerated rice genetic improvement, and laid a foundation for studying genomes of other crops such as *Zea mays* (Schnable et al., 2009) and *Triticum aestivum* (International Wheat Genome Sequencing Consortium [IWGSC], 2014). With the development of sequencing technology, the time required for sequencing has largely decreased while the sequencing quality has greatly improved, therefore resulting in more high-quality reference genomes of cultivated rice varieties such as MH63, ZH97, and R498 (Zhang et al., 2016, 2018; Du et al., 2017). The focus of rice research has also been gradually turned to elucidate biological characteristics and evolution processes and to analyze gene functions and related biological issues at the genomic level, as well as to identify genes related to important agronomic traits such as high yield, high quality, and stress resistance (Huang et al., 2010, 2011, 2015; Xun et al., 2012; Wei et al., 2014; Yano et al., 2016).

At present, numerous genes related to important agronomic traits (e.g., grain size) have been located and cloned, such as GS3 (Fan et al., 2006; Mao et al., 2018), GL3.1 (Qi et al., 2012), DEP1 (Huang et al., 2009), GW2 (Song et al., 2007), *qSW5* (Shomura et al., 2008), *GW8* (Wang et al., 2012b), and *GS5* (Li et al., 2011). Although the genome assembly of weedy rice WR04-6 has been constructed (Sun et al., 2019), the progress of identifying genes from weedy rice and the functional genomics research remains hindered due to a lack of more high-quality reference genomes. Generally, the morphological characteristics of weedy rice is between wild rice (*O. rufipogon*) and cultivated rice (*O. sativa* L.) (Sun et al., 2013; Cui et al., 2016). Our previous taxonomic study showed that LM8 is a low heterozygous weedy rice germplasm. The plants are homozygous and can be inherited stably that is characterized by very small grains. To discover genes from weedy rice germplasms of LM8, we constructed a genetic map based on whole-genome sequencing of a F_2_ population derived from the cross between LM8 and a cultivated rice variety. In combination with the phenotypic data of 12 important agronomic traits collected from the F_2_ population, we also tried to identify some new genes from the weedy rice. Moreover, to clarify the genomic characteristics from weedy rice germplasms of LM8, we generated a high-quality genome assembly of LM8 based on the Nanopore sequencing technology and characterized the LM8 genome to reveal its evolutionary relationship, which broadens our understanding of weedy rice at the genomic level. Based on our study, we found that the combination of genetic map and genome map is critical to quickly discover candidate genes such as plant-type, panicle-type, and gain-size in weed rice.

## Materials and Methods

### Plant Materials

The weedy rice LM8 was obtained from the China National Genebank. It shows erect and compact architecture similar to cultivated rice and harbors typical characteristics, such as small grain size and black hull. The cultivated rice variety Shen 08S was provided by the Anhui Academy of Agricultural Sciences. A F_2_ population (1229 samples) was obtained from a cross between LM8 and Shen 08S and was planted in the experimental fields under natural growth conditions in Nanning, Guangxi Autonomous Region, China. In this study, the F2 population were collected from one F1. Fresh and healthy leaves were collected at seedling stage and stored at 80°C for subsequent genomic DNA extraction.

### Population Sequencing and Genetic Map Construction

Fresh leaves of randomly selected 199 samples of the F_2_ population and their parents (LM8 and Shen 08S) were used to extract genomic DNA with the cetyltrimethylammonium bromide (CTAB) method. The Illumina PE150 libraries were constructed according to the manufacturer’s instructions and sequenced on an Illumina HiSeq X Ten platform. The two parental genotypes were sequenced at a higher depth (20 × coverage) to obtain 10 Gb data each, and F_2_ individuals were sequenced at a lower depth (∼ 10 × coverage) to obtain 5 Gb data each. Low-quality reads were removed to obtain clean reads, which were then mapped to the LM8 genome (LM8_v1) using BWA (mem -t 4 -k 32 -M -R) (Li and Durbin, 2009). SAMtools (sort rmup) (Li et al., 2009) was used to convert and sort the mapping results and to remove PCR duplicate reads. The clean reads of each F_2_ individual that passed the quality control were mapped to the reference genome (LM8_v1) for haplotype-based SNP calling. Development of polymorphic markers was performed by GATK (McKenna et al., 2010) for SNP identification and genotyping, and a total of 2,373,849 SNP markers were obtained. Then, these SNP markers were filtered by removing abnormal bases, abnormal genotypes, incomplete coverage markers, and segregation distortion markers, and were sorted into LGs (Yang et al., 2018). After filtering, 10,739 SNP markers were cluster into 12 LGs using JoinMap v4.1 (Mapping algorithm—ML Mapping, Regression mapping—Kosambi’s) (Stam, 1993).

### Phenotypic Evaluation of the F_2_ Population

We collected the main culm of plant individuals at 25 days after heading to measure the plant height (PH), tillering number (TN), flag leaf length (FLL), and flag leaf width (FLW) using a ruler. At maturity, the main panicles of plant individuals were harvested to measure panicle length (PL) using a ruler, and the primary branch number (PB) and secondary branch number (SB) (Ma et al., 2016) were recorded. The filled grains were used to calculate the grain length (GL), grain width (GW), grain thickness (GT), length width ratio (LWR), and thousand-grain weight (TGW) using an automatic seed analyzer with three replicates (Wanshen Detection Technology, Hangzhou, China). The analysis of variance (ANOVA) and correlations of phenotypic characteristics collected from the F_2_ population were conducted in R v3.6.2 (Langfelder and Horvath, 2012).

### QTL Mapping and Candidate Gene Prediction

QTL mapping was conducted using a permutation test (*n* = 1,000) in MapQTL6.0 with the composite interval mapping method to determine the limit of detection (LOD) value of each phenotype (Ooijen et al., 2009). Then the CIM mapping method in Win QTL Cartographer v2.5 software was used to locate the QTL position, contribution rate, and additive effect (Wang et al., 2012a). The 99% confidence interval of a QTL were determined as a candidate region, in which genes harbored non-synonymous coding mutations, premature or extended termination mutations were regarded as functional genes.

### Genome Library Construction and Sequencing

Genomic DNA was extracted from the fresh leaves of LM8 using Genomic kit (13343, Qiagen, Germany). Total RNA was extracted from five different tissues (root, leaf, stem, flower, and spike) by using the TRNzol Universal Total RNA extraction Kit (DP424, Tiangen, China). The total RNA was reserve transcribed into cDNA using SMARTer PCR cDNA Synthesis Kit (634926, Takara, China). PCR was performed using PrimeSTAR GXL DNApolymerase (R050A, Takara, China). The purity, concentration, and integrity of DNA and RNA were determined using NanoDrop™ One UV-Vis spectrophotometer (Thermo Fisher Scientific, United States), Qubit^®^ 3.0 Fluorometer (Invitrogen, United States) and Agilent 2100 Bioanalyzer (Agilent technologies, United States).

A library for Illumina paired-end sequencing with an insert size of 350–500 bp was constructed and sequenced on an Illumina HiSeq X ten platform (Illumina, San Diego, CA, United States). Oxford Nanopore library preparation was conducted according to the manufacturer’s instruction (13343, Qiagen, Germany) and sequenced on a PromethION platform (Oxford Nanopore Technologies, Oxford, United Kingdom). Fresh young leaves were vacuum-infiltrated with formaldehyde solution and used for cross-link action. The Hi-C library was prepared following the manufacturer’s protocol and sequenced on an Illumina HiSeq X ten platform. SMRTbell library of RNA-seq was constructed from a pooled cDNA sample of five different tissue (root, leaf, stem, flower, and spike) using SMRTbell template prep kit 2.0 (100222300, Pacific Biosciences, United States) and sequenced on a PacBio Sequel sequencer (Pacific Biosciences, Menlo Park, United States) to obtain full-length transcriptome data.

### Genome Assembly

The Illumina short reads were filtered using fastp v0.20.0 with default parameters (Chen et al., 2018). The abundance of 17 nt K-mers (-C -m 17 -s 400M) was used to estimate the genome size and heterozygous rate (Marçais and Kingsford, 2011; Liu et al., 2013; Koren et al., 2017). Correction of long reads generated from the Oxford Nanopore PromethION platform and *de novo* assembly were performed by *NextDenovo* v1.1.1 (read_cuoff = 2 k, seed_cutoff = 23 k, blocksize = 1 g, pa_raw_align = 20, pa_correction = 35) and *SMARTdenovo* (-e dom -J 5000 -k 17) (Loman et al., 2015; Cali et al., 2018). The Illumina short reads were mapped to the initial sequence assembly using BWA v0.7.12-r1039 with default parameters, which was then iteratively polished with three rounds of correction using NextPolish v3.0.1 (-max_depth 100 cluster_optons = -w n -l vf = {vf} -q all.q -pe smp {cpu} genome_size = auto) (Walker et al., 2014; Hu et al., 2020). Purge Haplotigs software was used to generate a contig-level assembly with only one copy of each of the contigs from heterozygous regions. The completeness of the draft genome was assessed by BUSCO v3 with the embryophyta_odb9 database (Simão et al., 2015).

Ultra-high-molecular-weight (uHMW) DNA (DNA length > 250 kb) were extracted using Bionano Prep Plant DNA Isolation Kit (80003; Bionano Genomics, United States) according to the manufacturer’s instructions. uHMW DNA molecules were labeled with the DLE-1 enzyme and loaded onto a Saphyr Chip and scanned for images on a Bionano Saphyr system (Bionano Genomics, San Diego, CA, United States). The raw molecules generated were quality-controlled and filtered (molecules with a size < 150 kb were removed). An optical map was generated using Bionano Solve package v3.4. The generated optical map was used to construct scaffolds using the Hybrid Scaffold pipeline of Bionano Solve package v3.4 (CL.py -d -U -N 6 -y -i 3 -F 1 -a opt Arguments_non-haplotype_noES_noCut_saphyr.xml) and Bionano Access v1.5.2 (Bionano Genomics, San Diego, CA, United States) with a more stringent (1e-13) merging *p*-value threshold (Xiao et al., 2007; Reisner et al., 2010; Mostovoy et al., 2016). The Hi-C raw reads were filtered by fastp v0.12.6 with default parameters and then mapped to the scaffolds with Bowtie2 (Langmead and Salzberg, 2012; Chen et al., 2018). We used Lachesis (ligating adjacent chromatin enables scaffolding *in situ*) to cluster, order, and anchor scaffolds onto the chromosomes (Burton et al., 2013; Dudchenko et al., 2017).

### Annotation of Genome

The repeat sequences and elements were annotated by a combination of *de novo* and homology-based methods. LTR_FINDER (Haas et al., 2008) and RepeatModeler (Haas et al., 2003) were used to generate a dataset of repetitive sequences with default parameters. This dataset was BLAST against the Plant Genome and Systems Biology (PGSB) repeat element database to classify the repeats (Spannagl et al., 2016), and then RepeatMasker was employed to annotate these repeats based on the Repbase database (Bao et al., 2015). Further, tandem repeats finder software was used to identify tandem repeats (Benson, 1999).

The protein-coding genes of the LM8 genome were predicted through a comprehensive strategy that combined results obtained from *de novo*, homology-based, and transcriptome-based predictions. Augustus was used for *de novo* prediction with Hidden Markov Model (Stanke et al., 2008). Homologous proteins from six plant genomes (*Arabidopsis thaliana*, *O. sativa*, *Zea mays*, *Hordeum vulgare*, *Physcomitrella patens*, and *Triticum aestivum*) were downloaded from Ensembl plants^1^ and used for homology-based prediction by GeMoMa (Jens et al., 2016). The non-redundant full-length transcripts obtained from the PacBio Sequel platform were aligned to the LM8 genome assembly for transcriptome-based prediction using PASA (Haas et al., 2003).

Gene structures were determined based on a combination of results from the three prediction methods using EvidenceModeler (Haas et al., 2008). Functional annotation of protein-coding genes was achieved by BLASTP searches against the Swiss-Prot database (Stanke and Waack, 2003). Protein domains were annotated by searching against the InterPro database using InterProScan (Zdobnov and Apweiler, 2001; Hunter et al., 2009). Non-coding RNA genes, including miRNA, snRNA, and rRNA genes were predicted according to the Rfam database, while tRNA genes were identified using tRNAscan-SE (Lowe and Eddy, 1997; Griffiths-Jones et al., 2005). The completeness of the predicted gene set was assessed by BUSCO v3 with the embryophyta_odb9 database (Benson, 1999).

### Collinearity Analysis

Protein sequences of LM8, *japonica* var. Nipponbare, and *indica* var. R498 were aligned by BLASTP v2.6.0 with default settings. Syntenic gene blocks within the genome were detected by MCScanX (Wang et al., 2012c) and visualized using the jcvi python module.

### Identification of Gene Families

Gene family identification was performed across LM8 (*O. sativa* f. *spontanea*), *O. aus* (AUS), 5 cultivated rice varieties, and 11 wild rice species. The 5 cultivated rice varieties included *O. sativa* ssp. *indica* (IND), *O. sativa* ssp. *japonica* (JAP), *O. sativa* ssp. *indica* var. Minghui63 (MH63), *O. sativa* ssp. *indica* var. Zhenshan97 (ZS97), *O. sativa* ssp. *indica* var. Shuhui498 (R498). The 11 wild rice species consisted of *O. glaberrima* (GLA), *O. barthii* (BAR), *O. glumaepatula* (GLU), *O. meridionalis* (MER), *O. rufipogon* (RUF), *O. nivara* (NIV), *O. longistaminata* (LON), *O. punctata* (PUN), *O. brachyantha* (BRA), *O. rufipogon* var. JX-6 (JX-6), and *O. rufipogon* var. Z59 (Z59). PUN and BRA belong to the BB and FF genomes, respectively, while the others belong to the AA genome. Across all species, the longest transcript of each gene was used in further analyses. Orthologous and paralogous gene clusters were identified using BLASTP (-e 1e-5 -F F). Clustering analysis of protein sequences from the 18 *Oryza* genomes was conducted with OrthoMCL (Li et al., 2003).

### Phylogenetic Analysis

Multiple sequence alignments of the protein-coding sequences of the 4,241 single-copy orthologous genes obtained from the above analysis these protein sequences were performed by MAFFT (Katoh and Standley, 2013). Phylogenetic relationships were resolved using RAxML (-m GTRGAMMA -p 12345 -T 8 -f b -t -z) among these 18 *Oryza* genomes with all single-copy genes concatenated into an ultra-long aligned sequence, where *O. brachyantha* was designated as an outgroup (Stamatakis, 2014). Divergence times were estimated by MCMCtree (Puttick, 2019) with parameters of “RootAge ≤ 0.21, rgene gamma = 23.52254, burnin = 100,000, sampfreq = 100, nsample = 50,000, model = 7” in the PAML package (Nikolau et al., 2003) based on a known divergence time (∼ 0.4 Mya) between *O. nivara* and *O. rufipogon*.

### Expansion and Contraction of Gene Families

A random birth-and-death model was used to estimate changes in gene families between the ancestor and each species using CAFE with conditional likelihoods as the test statistics (-p 0.05 -t 10 -r 10000 lambda -s) (De Bie et al., 2006). A probabilistic graphical model (PGM) was used to calculate the probability of transitions in each gene family, and then all the gene families were classified into three types (expanded, contracted, and unchanged). Finally, GO enrichment was performed for further functional analysis of the expanded genes.

### Positive Selection Analysis

All orthologous genes identified in the LM8 genome were tested for positive selection. The phylogenetic tree generated by RAxML was used as the input, and the branch-site test was conducted with CodeML (model = 2, NSsites = 2, fix_omega = 0, fix_omega = 1, omega = 1) in the PAML package (Yang, 2007). Genes under positive selection were determined based on the likelihood ratio test (*P* < 0.01).

## Results

### Genetic Map Construction and QTL Analysis With a F_2_ Population

**FIGURE 1:**
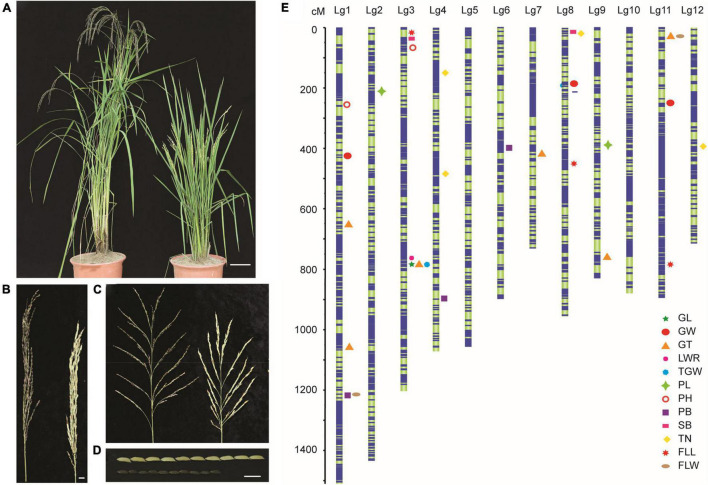
Agronomic characteristics of LM8 and Shen 08S (parents) and the genetic map constructed based on the F_2_ population. **(A)** Plant height of LM8 (right) and Shen 08S (left). Bar = 10 cm. **(B)** Panicle length of LM8 (right) and Shen 08S (left). Bar = 0.5 cm. **(C)** Branches of LM8 (right) and Shen 08S (left). Bar = 2 cm. **(D)** Grain size of LM8 (bottom) and Shen 08S (above). Bar = 1 cm. **(E)** Genomic locations of 12 agronomic trait associated QTLs are illustrated on the 12 linkage groups. PH, plant height; TN, tillering number; FLL, flag leaf length; FLW, flag leaf width; PL, panicle length; PB, primary branch number; SB, secondary branch number; GL, grain length; GW, grain width; GT, grain thickness; LWR, length to width ratio; TGW, thousand-grain weight.

To further understand the mechanism of LM8 genome variation in its special grain formation, a F_2_ population was generated from the cross between LM8 and a cultivated rice variety Shen 08S. The two parents, LM8 and Shen 08S, showed obvious differences in plant height, panicle length, and grain size (Figure 1 and Supplementary Table 1). We sequenced the genome of F_2_ individuals as well as that of the two parents. A total of 10,739 high-quality SNPs were obtained and used to generate a genetic map. The total genetic distance of the constructed genetic map was 12,171.13 cM, and the average genetic distance between two SNPs was 1.13 cM (Figure 1). The SNPs were distributed throughout the 12 linkage groups (LGs) with the highest SNP number (1,754) occurring on LG1 (1,510.48 cM total size) and the lowest (523) on LG12 (713.79 cM total size). Collinearity analysis showed that the genetic map had strong collinearity (99.69%) with the reference genome sequence (Supplementary Figure 1), and the sources of most segments in F_2_ individuals were consistent according to the monomer source analysis. These results suggest that the constructed genetic map is of high-quality and suitable for further analyses.

**FIGURE 2:**
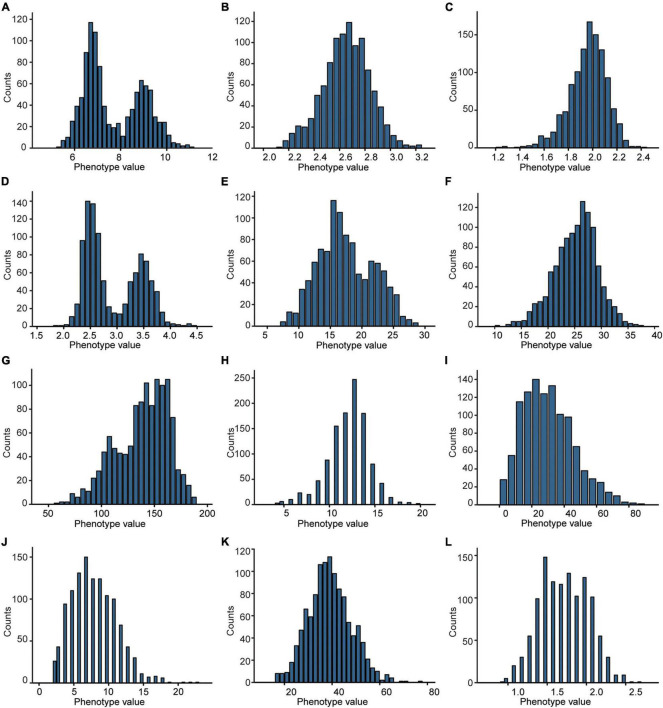
Frequency distribution of the 12 agronomic traits in the F_2_ population. **(A)** GL, grain length. **(B)** GW, grain width. **(C)** GT, grain thickness. **(D)** LWR, length to width ratio. **(E)** TGW, thousand-grain weight. **(F)** PL, panicle length. **(G)** PH, plant height. **(H)** PB, primary branch number. **(I)** SB, secondary branch number. **(J)** TN, tillering number. **(K)** FLL, flag leaf length. **(L)** FLW, flag leaf width.

Besides, combining the phenotypic data (Figure 2) obtained from the F_2_ population and the genetic map, we identified 31 quantitative trait loci (QTLs) with 607 genes related to 4 plant-type traits, 3 panicle-type traits, and 5 grain-size traits (Figure 1). Eight of the QTLs explaining more than 17% of the phenotypical variation were identified as major QTLs, which were located at 788.3–789.4 cM on chromosome 3 (chr3), 34.4–37.5 cM on chr11, 782.9–786.6 cM on chr3, 787.6–788.2 cM on chr3, 244.6–253 cM on ch11, 204.9–217.6 cM on chr2, 11.4–17.3 cM on chr8, and 33.6–38 cM on chr11 (Supplementary Table 2 and Supplementary Figure 2). Fourteen QTLs were identified to be associated with grain-size traits, including 1 for grain length (GL), 3 for grain width (GW), 6 for grain thickness (GT), 1 for length width ratio (LWR), and 3 for thousand-grain weight (TGW). These results would help in further detecting the genes from the weedy rice LM8.

### Identification of Candidate Genes Related to Grain Length

**FIGURE 3:**
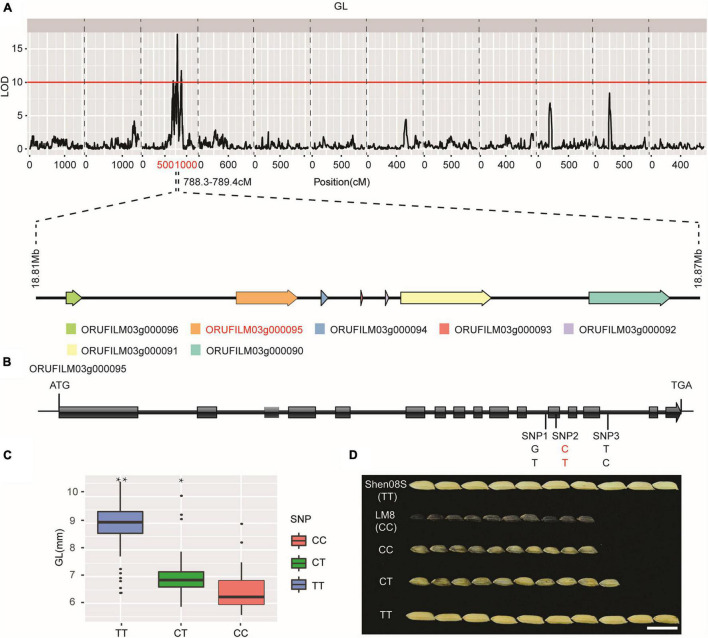
QTL for grain length and prediction of candidate genes. **(A)** QTL mapping results for grain length and seven putative genes (*ORUFILM03g000096*, *ORUFILM03g000095*, *ORUFILM03g000094*, *ORUFILM03g000093*, *ORUFILM03g000092*, *ORUFILM03g000091*, and *ORUFILM03g000090*) within the region. **(B)** Gene structure of *ORUFILM03g000095* (top), **(C)** grain length (GL) distribution in each genotype of *ORUFILM03g000095*, **(D)** corresponding GL phenotypes in F_2_ individuals (lower right). Asterisks is correlation between genotype and phenotype, *P < 0.05. **P < 0.01.

LM8 has evolved to form extremely small grains that may develop new elite rice varieties to study grain shape or yield related traits. Therefore, using LM8 as the material to discover genes related to grain size is practical to enrich rice resources. We conducted a correlation analysis among the grain-size traits QTLs, including grain length, grain width, grain thickness, length to width ratio, and thousand-grain weight. Significant positive correlations (*P* < 0.05) were observed among grain length, length to width ratio, and thousand-grain weight, indicating that grain length has significant impact on grain size (Supplementary Table 3 and Supplementary Figure 3). One QTL related to grain length was located at 788.3–789.4 cM on chr3, corresponding to a 60-kb interval harboring seven putative genes, which included 3 RAPdb annotated genes (*ORUFILM03g000091*, *ORUFILM03g000095*, *ORUFILM03g000096*) and 4 unknown function annotations (*ORUFILM03g000090*, *ORUFILM03g000092*, *ORUFILM03g000093*, *ORUFILM03g000094*) were important candidate genes controlling grain length (Figure 3 and Supplementary Table 4).

*OsCLG1* (Yang et al., 2021) mediate ubiquitin ligase to regulate grain length. Therefore, the candidate genes among seven candidate genes, *ORUFILM03g000095* is a homologous gene to *Os03g0427900* of *Nipponbare* and belongs to the U-box protein gene family, in which a U-box domain acts as a ubiquitin ligase to participate in protein degradation during the cell cycle and morphological development (Sharma and Taganna, 2020; Yang et al., 2021). To further investigate the molecular basis of the small grain phenotype in LM8, we analyzed the sequence of *ORUFILM03g000095* gene from LM8, Shen 08S, and their progenies and revealed a C-T SNP site, located in the 12th exon 5,339 bp downstream of the ATG start site (Figure 3). Grain length in the F_2_ individuals of LM8 and Shen 08S displayed a clear pattern with an order of TT > CT > CC (*P* < *0.01*; Figure 3). *ORUFILM03g000095* genotypes were significantly correlated to the grain length variation, suggesting that this locus plays an important role in grain size regulation. Our results suggest that *ORUFILM03g000095* are possible candidate genes controlling grain length. However, the underlying mechanisms of how this gene regulate grain formation remain elusive and need to be further explored.

### Genome Assembly and Annotation

There are major differences between the morphology of weedy rice and cultivated rice (*O. sativa* L.). The current research on cultivated rice is relatively clear, but the research on weedy rice does not yet have a reference genome with high assembly quality. To clarify the genome characteristics of the F2 population parent (weed rice LM8), we assembled a high-quality genome. Before assembly, *SOAPdenovo* was used for pre-assembly. K-mer analysis (*k* = 17) estimated its genome size to be around 362.7 Mb, with a moderate heterozygous rate of 0.20% (Supplementary Figure 4). However, the completeness and quality of the assembly are not ideal if the genome is assembled using the second-generation sequencing data alone. Thus, the LM8 genome was sequenced and assembled by applying a combination of diverse technologies, including Oxford Nanopore long-read sequencing, Illumina short-read sequencing, Bionano optical mapping, and Hi-C technology (Supplementary Table 5 and Supplementary Figure 5). A total of 77.2 Gb raw data (sequencing depth 100x) were collected from Oxford Nanopore long-read sequencing, which were then self-corrected, filtered, and polished to generate the final dataset (57.3 Gb) for genome assembly (Table 1 and Supplementary Figure 6). The contig-level assembly (LM8_contig) comprised 375.3 Mb, with a contig N50 of 17.9 Mb (Table 1 and Supplementary Table 6). Approximately 98.1% ubiquitous genes in embryophyte were detected by the Benchmarking Universal Single-Copy Orthologs (BUSCO) analysis (Supplementary Table 7), indicating that the assembled contig was of high-completeness.

**TABLE 1:** Summary of the sequencing, assembly, and annotation of the LM8 genome.

Stat type	Number
Assembled genome size (Gb)	77.2
Contig N50 (Mb)	17.9
Scaffold N50 (Mb)	30.5
Longest scaffold (Mb)	31.3
Anchored to chromosome (Mb)	375.8
Number of predicted protein-coding genes	36,561
Average gene length (bp)	3545.1
Average CDS length (bp)	1129.6
Average exons number per gene	4.4
Average exon length (bp)	255
Average intron length (bp)	705
Number of rRNAs	81
Number of snRNAs	772
Number of miRNAs	2,551

Next, using 476.2 Gb of molecules (> 150 kb) collected from Bionano Saphyr system, we generated an optical map for the LM8 genome, with a total size of 370.3 Mb and an N50 of 24.2 Mb. With the aid of this optical map, we further assembled LM8_contig into scaffolds (LM8_scaffold), with a total size of 375.8 Mb and a scaffold N50 of 24.1 Mb (Supplementary Table 6). After applying high-throughput chromosome conformation capture (Hi-C) data to orient, order, and phase these scaffolds, a total of 375.3 Mb sequences (99.85%; Supplementary Table 8) were anchored onto the 12 chromosomes and the final chromosome-level genome assembly (LM8_v1) was obtained. The Hi-C heatmap separated different chromosomes and showed that the interaction intensity in the diagonal-position was higher than that in the off-diagonal-position (Supplementary Figure 7). BUSCO analysis showed that 97.9% of the core embryophyte genes were complete in the LM8 genome assembly (Supplementary Table 7). In addition, 87.1% (31,810) of the predicted genes were expressed according to the transcriptome data. The above results suggest that the LM8 genome assembly is of high-quality and -completeness.

**FIGURE 4:**
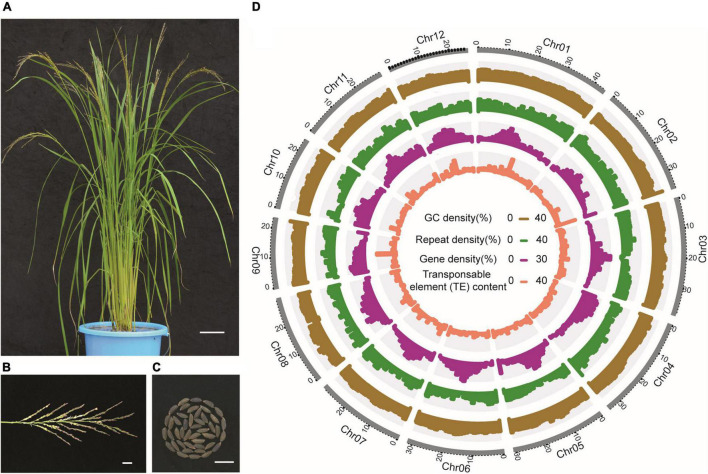
The morphology and genome features of LM8. **(A)** Whole plants, bar = 10 cm. **(B)** Panicles, bar = 2 cm. **(C)** Grains, bar = 1 cm. **(D)** Genome features of LM8. Circles from inside to outside are transposable element (TE) content, repeat density, gene density, and GC density.

Repeat annotation results showed that 47.72% of the LM8 genome is composed of repetitive sequences, including 26.87% retrotransposons and 20.85% DNA transposons. About 94.08% of retrotransposons are long terminal repeats (LTRs), accounting for 25.28% of the genome. The two most frequent types of LTRs are *Copia* and *Gypsy*, accounting for 2.99 and 19.62%, respectively (Figure 4 and Supplementary Table 9). Besides, through a comprehensive strategy combining results obtained from *de novo*, homology-based, and transcriptome-based prediction, 36,561 protein-coding genes were annotated in the LM8 genome. These protein-coding genes have an average length of 3,545.1 bp, an average coding sequence length of 1,129.6 bp, an average exon length of 255.2 bp, an average intron length of 705.1 bp, and an average exon number per gene of 4.4 (Table 1). Among these annotated genes, 34,773 (95.91%) were functionally annotated by at least one of the Swiss-Prot, KEGG, and InterPro databases (Supplementary Table 10). In addition, homology-based annotation of non-coding RNAs (ncRNAs) predicted 2,551 microRNAs (miRNAs), 81 ribosomal RNAs (rRNAs), and 772 small nuclear RNAs (snRNAs; Supplementary Table 11).

### Comparative Analysis

**FIGURE 5:**
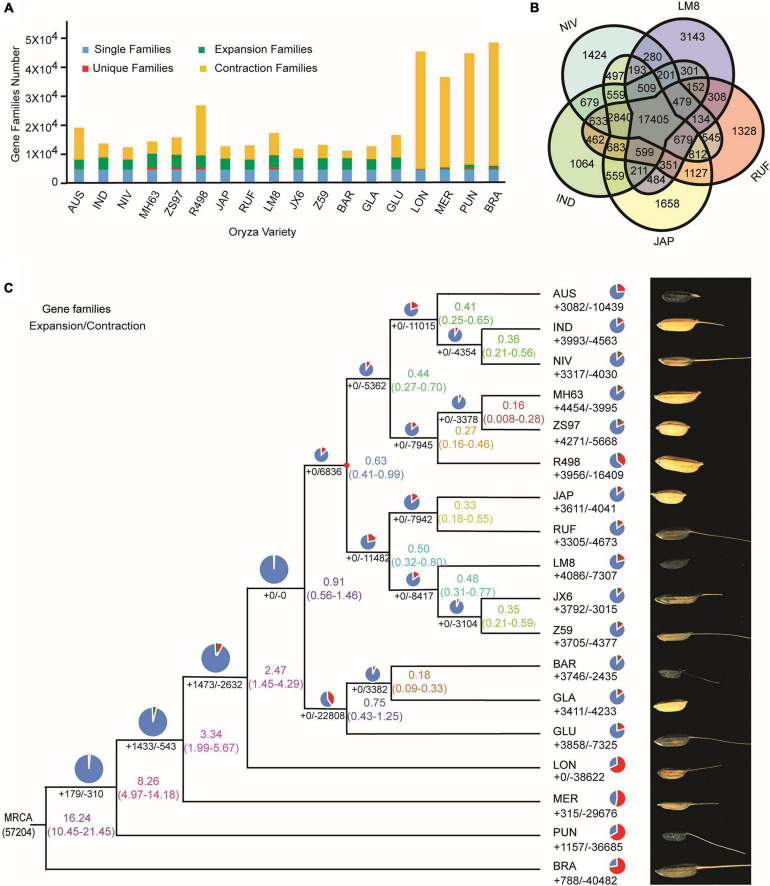
Comparative genomics analyses of LM8 with other *Oryza* genomes. **(A)** Statistics of gene families in 18 *Oryza* genomes. **(B)** Core and dispensable genes from five reference genomes. The numbers in the species section and overlapping section indicate the numbers of specific and shared gene families, respectively. IND, *O. sativa* ssp. *indica*. JAP, *O. sativa* ssp. *japonica*. RUF, *O. rufipogon*. NIV, *O. nivara*. and LM8, *O. sativa* f. *spontanea*. **(C)** Phylogenetic relationships and grain phenotypes of LM8 and other *Oryza* genomes. Pie charts represent total gene families, consisting of contracted gene families (red), expanded gene families (green), and unchanged gene families (blue). The numbers of genes in expanded (+) and contracted (–) gene families in each rice variety are shown with the rice variety name farthest to the right. The lineage divergence times are indicated on the nodes and nodes marked in red are known fossil time points. *O. brachyantha* was used as the outgroup. MRCA, most recent common ancestor. AUS, *O. aus*. IND, *O. sativa* ssp. *indica*. JAP, *O. sativa* ssp. *japonica*. GLA, *O. glaberrima*. BAR, *O. barthii*. GLU, *O. glumaepatula*. MER, *O. meridionalis*. RUF, *O. rufipogon*. NIV, *O. nivara*. LON, *O. longistaminata*. PUN, *O. punctata*. BRA, *O. brachyantha*. JX-6, *O. rufipogon* var. JX-6. Z59, *O. rufipogon* var. Z59. MH63, *O. sativa* ssp. *indica* var. Minghui63. ZS97, *O. sativa* ssp. *indica* var. Zhenshan97. R498, *O. sativa* ssp. *indica* var. Shuihui498. and LM8, *O. sativa* f. *spontanea*.

**FIGURE 6:**
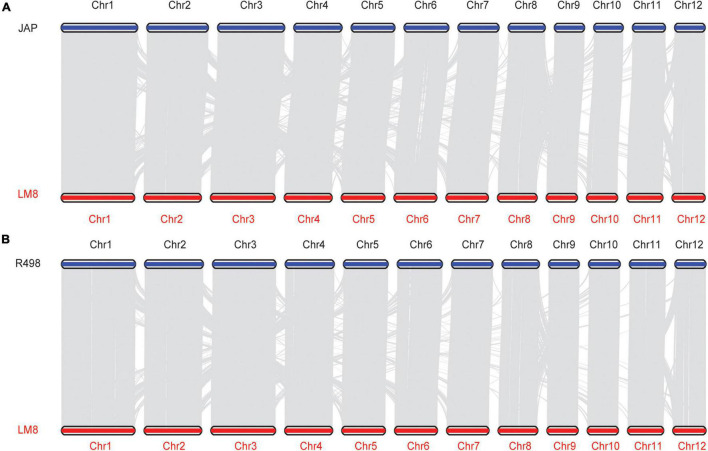
Collinearity between LM8 and two cultivated rice (JAP and R498) genomes. **(A)** Collinearity between JAP and LM8. **(B)** Collinearity between R498 and LM8.

To reveal the evolutionary relationship of the weedy rice LM8, 4,241 single-copy orthologous genes of LM8 and those from other 17 *Oryza* genomes were used to construct a phylogenetic tree by the maximum-likelihood (ML) method (Figure 5 and Supplementary Table 12 and Supplementary Figure 8). The phylogenetic tree demonstrated that LM8 diverged from the ancestor *O. rufipogon* ∼ 0.32 million years ago (Mya; Figure 5) and was clustered into a group with *japonica*, indicating LM8 is more closely related to *japonica* compared to *indica*. Additionally, genome collinearity analyses conducted between LM8 and two cultivated rice varieties revealed that the LM8 genome had more collinear genes with *japonica* var. Nipponbare (47,439/78,939; 60.1%) than *indica* var. R498 (34,750/74,110; 46.89%; Figure 6 and Supplementary Figure 9). Collectively, we speculate that LM8 belongs to *japonica*-type weedy rice.

**FIGURE 7:**
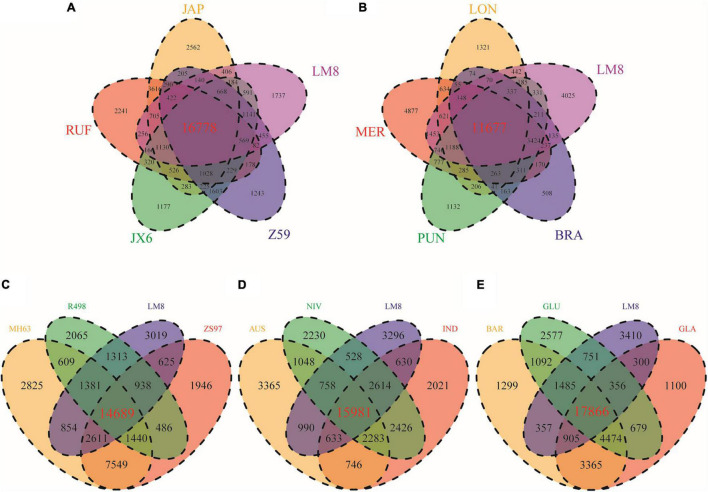
Venn diagrams showing specific and shared gene families between LM8 and other *Oryza* genomes in the same cluster of the evolution tree. **(A)** JAP, RUF, JX6, Z59 and LM8. **(B)** LON, MER, PUN, BRA and LM8. **(C)** MH63, R498, ZS97 and LM8. **(D)** AUS, NIV, IND and LM8. **(E)** BAR, GLU, GLA, and LM8. AUS, *O. aus*. IND, *O. sativa* ssp. *indica*. JAP, *O. sativa* ssp. *japonica*. GLA, *O. glaberrima*. BAR, *O. barthii*. GLU, *O. glumaepatula*. MER, *O. meridionalis*. RUF, *O. rufipogon*. NIV, *O. nivara*. LON, *O. longistaminata*. PUN, *O. punctata*. BRA, *O. brachyantha*. JX-6, *O. rufipogon* var. JX-6. Z59, *O. rufipogon* var. Z59. MH63, *O. sativa* ssp. *indica* var. Minghui63. ZS97, *O. sativa* ssp. *indica* var. Zhenshan97. R498, *O. sativa* ssp. *indica* var. Shuihui498. and LM8, *O. sativa* f. *spontanea*.

By comparing LM8 with four other rice species including *O. nivara* (NIV), *O. sativa* ssp. *indica* (IND), *O. sativa* ssp. *japonica* (JAP), and *O. rufipogon* (RUF), we found that 68.4% (17,403/25,430) of the gene families in LM8 were shared among all five species, while approximately 12.4% (3,143/25,430) were specific to LM8 (Figure 5). The closer the relationship indicated by the phylogenetic tree, the more the shared gene families (Figure 7). Among the 18 *Oryza* genomes, 2,875 unclustered genes and 672 unique genes were observed in the LM8 genome (Supplementary Table 12 and Supplementary Figure 8). The proteins encoded by these unique genes related to the formation of grain length, heading date and tillering number including serine/threonine-protein phosphatases (*ORUFILM03g000947*), photosystem II reaction center proteins (*ORUFILM08g001423*), and zinc finger MYM-type proteins (*ORUFILM03g000136*).

### Gene Family Analysis

**FIGURE 8:**
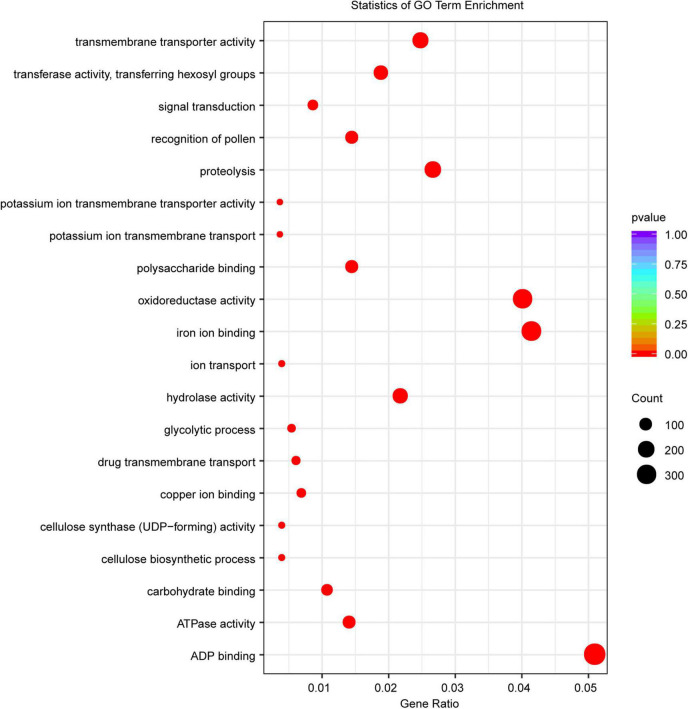
Statistics of the GO enrichment analysis of the expanded gene families. The *x*-axis represents the percentage of enriched genes to the total annotated genes. The *y*-axis indicates the entry of each enrichment category. The size of the dots corresponds to the number of enriched genes, and the color panel on the right indicates the *q*-value. The lower the value, the more significant it is.

Gene family expansion/contraction has been shown to be associated with domestication and ecological adaptation (Peng et al., 2019; Zeng et al., 2019). To characterize the LM8 genome, a genome-wide comparative genomics analysis was performed among 18 *Oryza* genomes (Supplementary Table 13). We assigned 36,561 LM8 genes to 25,430 gene families (Table 1 and Supplementary Table 14). Relative to the common ancestor of rice (*O. rufipogon*), 16.06% (4,086/25,430) expansion and 28.73% (7307/25,430) contracted gene families were observed (Figure 5 and Supplementary Table 14). The expansion gene families included 12793 expansion genes, of which 213 QTL mapping genes belonged to the expanded gene family. In the expanded gene families, Gene Ontology (GO) enrichment analysis revealed 295 GO terms involving biological process (BP), cellular component (CC), and molecular function (MP). Sixty-seven pathways were significantly enriched, including carbohydrate metabolic process, signal transduction, and cell growth (Figure 8). The significantly enriched genes may contribute to the adaptability of LM8 to complex environments during evolution. Meanwhile, we found 57 genes among the QTL mapping were detected by GO enrichment and enriched into 20 pathways including catalytic activity, proteolysis, and transmembrane transport protein activity (Supplementary Table 15). A total of 168 positive selection genes (PSGs) were identified and annotated to be auxin response proteins (e.g., *ORUFILM02g003288*), cell division control proteins (e.g., *ORUFILM01g004046*), and ubiquitin-protein ligase E3 UPL4 (e.g., *ORUFILM05g002772*), which may participate in the regulation of grain growth process and grain formation (Luo et al., 2013; Basunia et al., 2021). Nevertheless, whether these PSGs can explain the difference in the grain size need to be further explored.

## Discussion

With the development of sequencing techniques and corresponding analysis approaches, the sequencing speed and quality have greatly improved, while the cost has decreased tremendously, allowing a growing number of genomes to be sequenced and applied to related studies. The combination of a specific chromosome-level genome assembly and a high-density genetic map has been verified to be effective to map QTLs or locate genes associated with important agronomic traits (Luo et al., 2020) and has been widely applied to various important crops including cotton (Wang F. et al., 2020), peanut (Agarwal et al., 2018), *Cucumis melo* (Hu et al., 2018), pear (Li et al., 2019). In rice, Li et al. (2018) constructed a high-density genetic map through performing whole-genome resequencing and identified a candidate gene (*DEP1*) in determining panicle length. Later, Sun et al. (2019) constructed a genetic map and located a region on chr1 contributing to seed shattering, awn length, and plant height. In this study, we generated a chromosome-level genome assembly and constructed a high-density genetic map with the help of high-throughput sequencing approaches, we identified *ORUFILM03g000095* gene on chr3 that may regulate grain length (Figure 3). We have analyzed the candidate gene based on 3K genome data which is important research in rice genomics research (Wang et al., 2018; Wang C. et al., 2020), but the same haplotype as LM8 was not found in 3K data, so we did not further analyze it (Supplementary Table 16). This study would not only lay a foundation for rapid discovery of genes from weedy rice but also broaden the understanding of weedy rice utilization on rice genetic improvement. Large number of candidate genes were obtained in this study and those excellent gene could improve the breeding value of cultivated rice. Next step studying of the function of the candidate gene can use gene knockout, mutation analysis, overexpression analysis, genetic complementation, and other experiments to further verify whether the candidate gene can be used to improve cultivated rice.

The *Oryza* genus is generally believed to include 22 wild and 2 cultivated rice species based on morphological characteristics (Jacquemin et al., 2014). Asian cultivated rice (*O. sativa* L.), an important staple crop, is widely planted around the world and has formed extremely rich genetic diversity during the long evolutionary process. In *O. sativa*, the two subspecies (i.e., *indica* and *japonica*) differ in morphology, anatomical structure, physiological and biochemical characteristics, and genome sequence, and their origins remain controversial (Shinobu et al., 2002; Vaughan et al., 2007). The single-origin theory believes that *indica* and *japonica* both derived from *O. rufipogon* and diverged during the long-term domestication and artificial selection (Chang, 1976; Zhu and Ge, 2005). By contrast, the multi-origin theory believes that *indica* originated from *O. nivara* in eastern India, while *japonica* originated from *O. rufipogon* in the Yangtze River region of China (Oka, 1974; Londo et al., 2006; Huang et al., 2012; Sun et al., 2015), and the divergence between *indica* and *japonica* subspecies occurred 0.4 Mya (Kumagai et al., 2010; Chen et al., 2012). Our phylogenetic analysis showed that *O. nivara* and *O. rufipogon* were present in two separate branches, supporting the evolutionary model of multiple origins. LM8 was originated approximately 0.32 Mya and harbors morphological characteristics specific to wild rice such as shattering, hard glumes, and small grains (Figure 5). Thus, it could be concluded that LM8 is a kind of *japonica*-type weedy rice from a cross between *japonica* and wild rice, which confirmed the result of taxonomic study.

Chromosome-level genome assemblies may generally accelerate gene discovery in crops to improve yield, quality, and disease resistance (Rao et al., 2014; Qian et al., 2016; Bai et al., 2018). As genome assemblies of Asian cultivated rice varieties such as MH63, ZH97, and R498 become available, a large number of structural variations have been successfully obtained, which would have a wide-range impact on crop genetic improvement (Zhang et al., 2016; Du et al., 2017). For example, Zhang et al. (2014) assembled five AA-genome rice species and identified 14 PSGs that are closely related to rice flowering, development, reproduction, biotic and abiotic resistance through comparative genomics analyses. Although many genomes have been assembled in the *Oryza* genus, only one of them belongs to weedy rice (WRAH), which was used to discuss the origin of weedy rice (Sun et al., 2019). In this study, we reported another weedy rice (LM8) genome for the purpose of identifying genes. This chromosome-level genome assembly contains 672 unique genes specific to weedy rice compared with other 17 *Oryza* genomes (Figure 5). Besides, the comparison of the contig N50 (6.09 Mb in WRAH and 17.86 Mb in LM8) and sequence gaps (94 in WRAH and 25 in LM8; Table 1) between these two weedy rice genomes (Sun et al., 2019) indicates the high-quality LM8 genome assembly is able to serve as a reference for accelerating the identification of genes from weedy rice, thus improving the cultivated rice varieties.

## Data Availability Statement

The original contributions presented in the study are publicly available. This data can be found here: National Center for Biotechnology Information (NCBI) BioProject database under accession number PRJNA754271.

## Author Contributions

FL performed the experiments. FL and ZH conducted data analyses and wrote the manuscript. WQ contributed to construct of genetic population and experimental guidance. JW, YS, YCu, JL, JG, DLo, and WF contributed to help data analyses. DLi, BN, ZZ, YCh, and LZ contributed to the material preparation, collection, and measurement. QY and XZ designed the experiment and revised the manuscript. All authors read and approved the final manuscript.

## Conflict of Interest

The authors declare that the research was conducted in the absence of any commercial or financial relationships that could be construed as a potential conflict of interest.

## Publisher’s Note

All claims expressed in this article are solely those of the authors and do not necessarily represent those of their affiliated organizations, or those of the publisher, the editors and the reviewers. Any product that may be evaluated in this article, or claim that may be made by its manufacturer, is not guaranteed or endorsed by the publisher.

## Footnotes

1
http://plants.ensembl.org
