# Supplementary material for: High-Quality Genomes and High-Density Genetic Map Facilitate the Identification of Genes From a Weedy Rice
Source: Front Plant Sci. 2021 Nov 19;12:775051. doi: 10.3389/fpls.2021.775051 (PMC8639688; doi:10.3389/fpls.2021.775051)
Supplement: Supplementary file 1 [file Data_Sheet_1.docx]

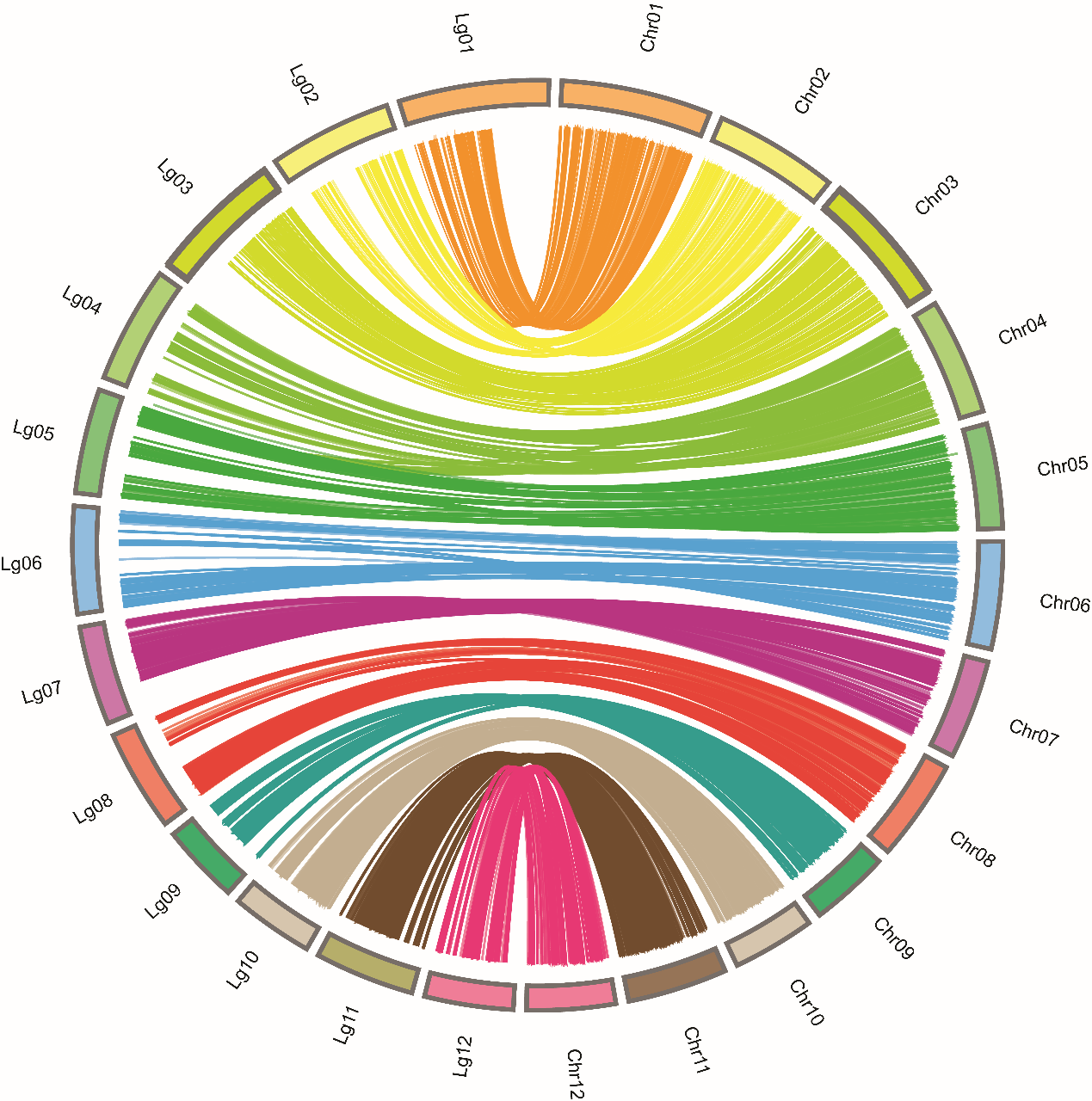


**Figure S1** Collinearity between the genetic map and genome assembly of LM8. Chr01-Chr12 represent the 12 chromosomes of the LM8 genome, and Lg01-Lg12 represent the 12 linkage groups in the genetic map.


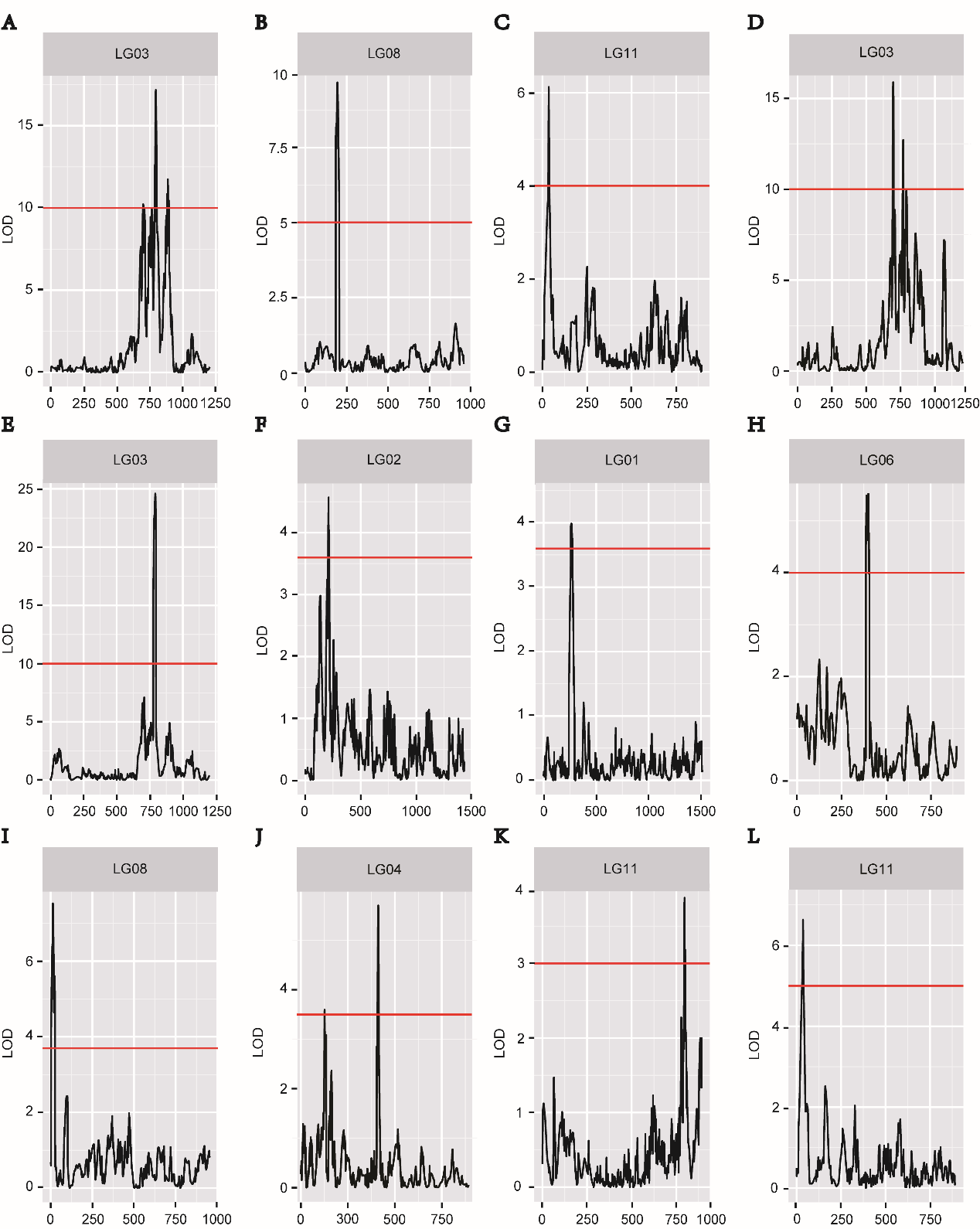


**Figure S2** QTL mapping of the 12 agronomic traits in the F_2_ population. LG represents linkage groups of the genetic map. (A) GL, grain length. (B) GW, grain width. (C) GT, grain thickness. (D) LWR, length to width ratio. (E) TGW, thousand-grain weight. (F) PL, panicle length. (G) PH, plant height. (H) PB, primary branch number. (I) SB, secondary branch number. (J) TN, tillering number. (K) FLL, flag leaf length. (L) FLW, flag leaf width.


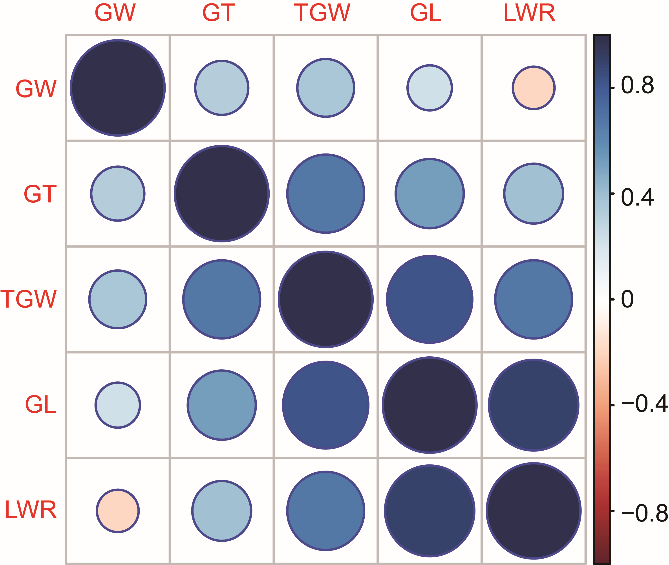


**Figure S3** Correlation analysis of the grain size traits. Correlation increases from 0 to 1 and 0 to -1, respectively. The abbreviations are the same as those in Figure S11.


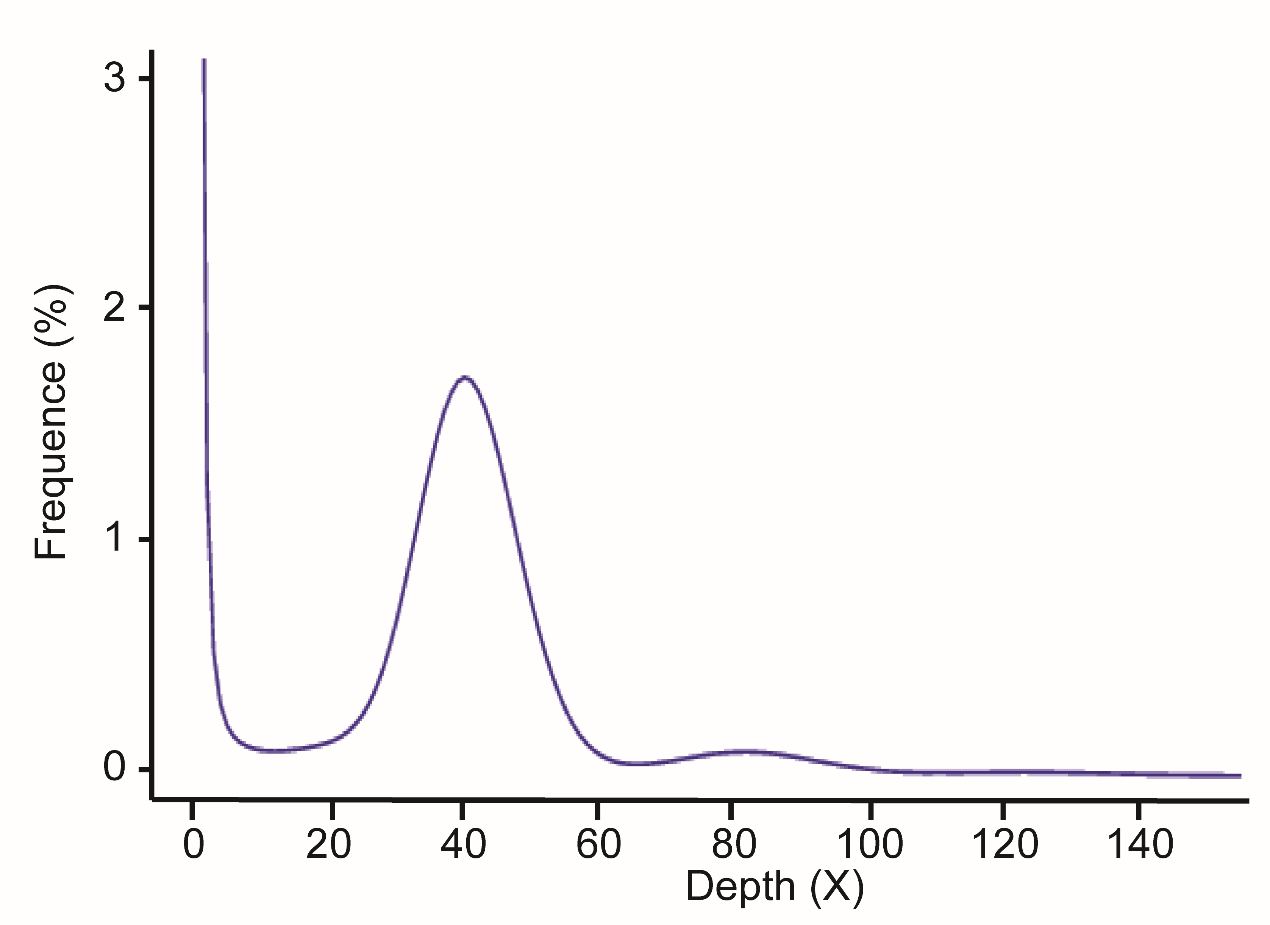


**Figure S4** Frequency distribution of the LM8 genome at Kmer =17.


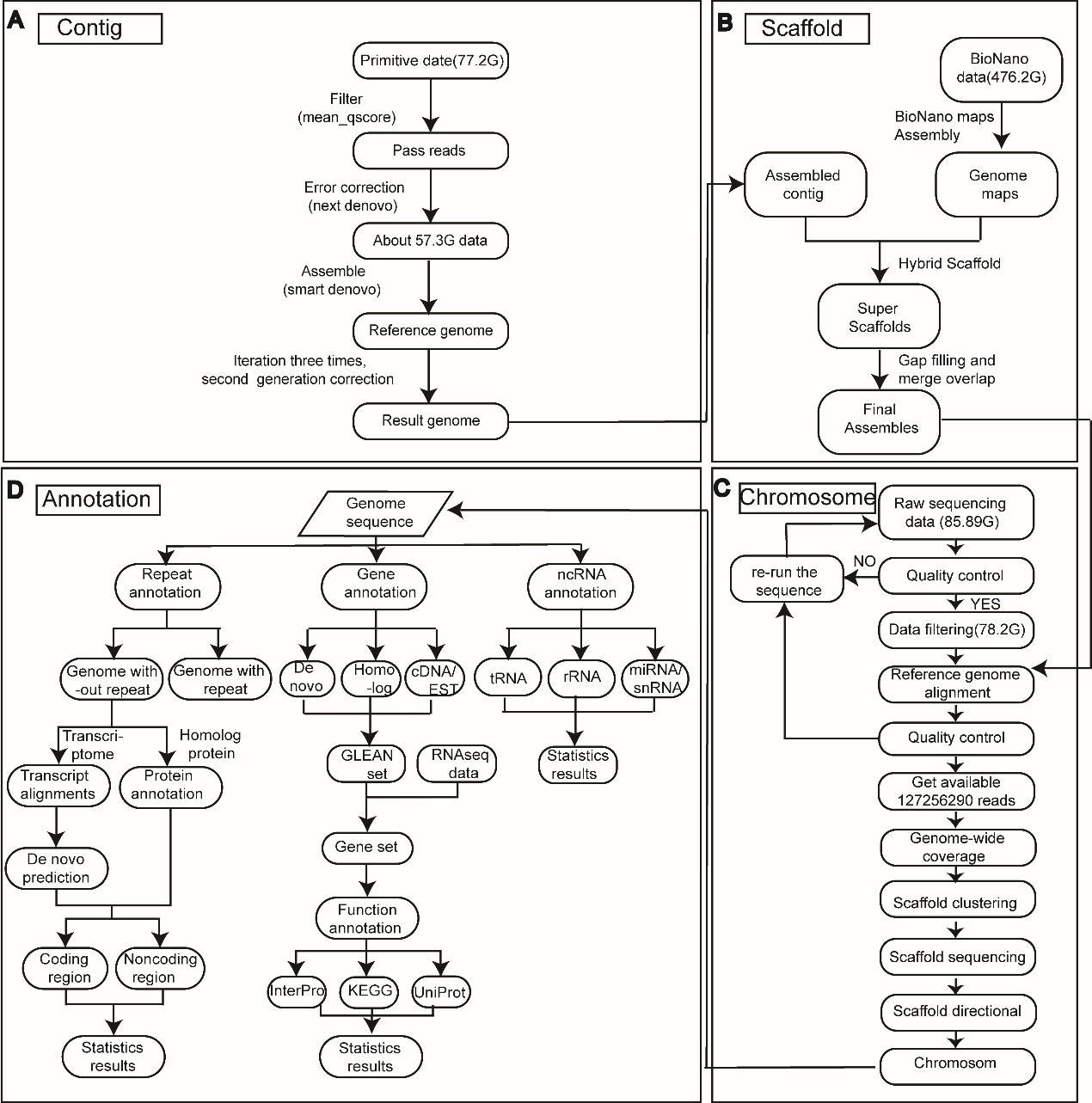


**Figure S5** Flowcharts of sequence assembly and annotation of the LM8 genome. (A) Contig assembly. (B) Scaffold assembly. (C) Chromosome assembly. (D) Genome annotation.


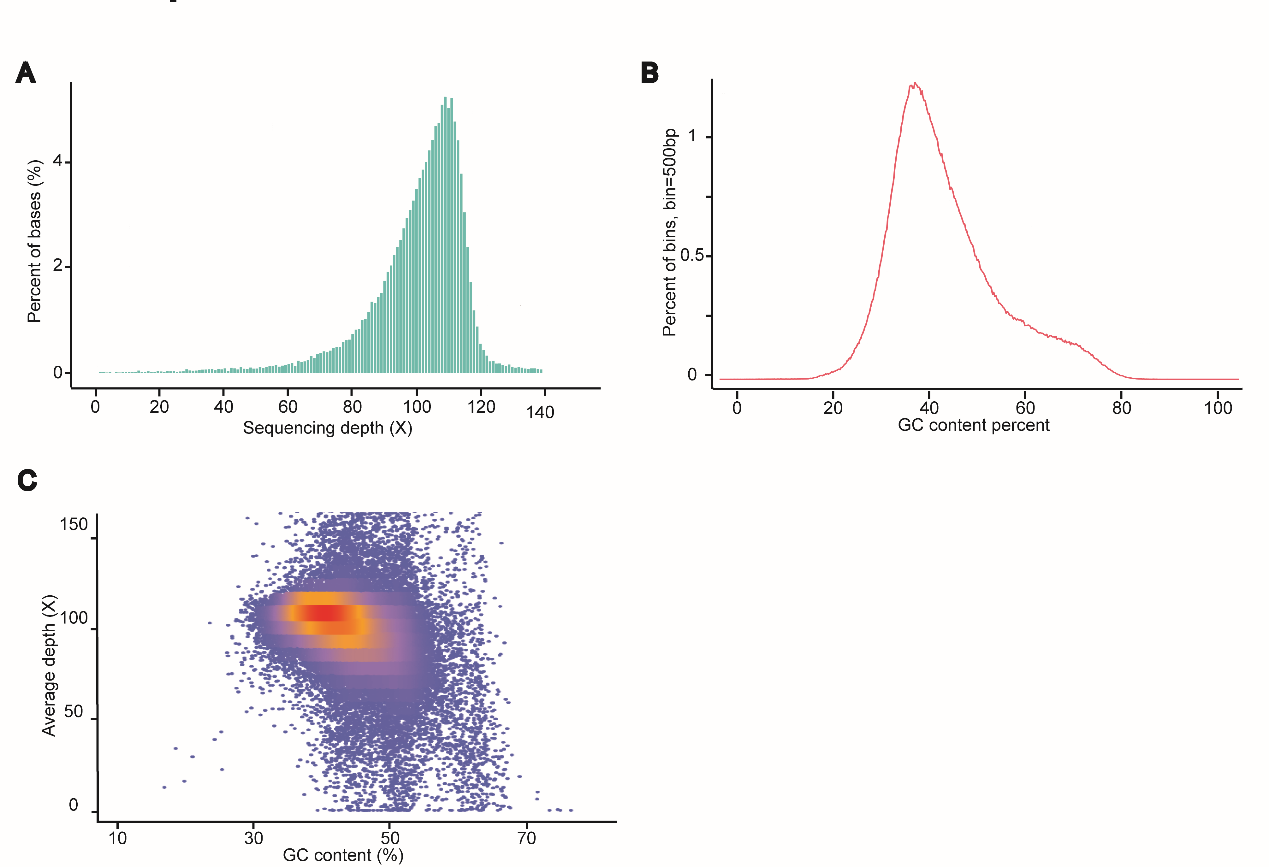


**Figure S6** Genomic characteristics of the LM8 genome. (A) Genome sequencing depth. The x-axis represents the sequencing depth that was counted in 5-kb windows. The y-axis represents the ratio of the number of bases corresponding to the sequencing depth to the total bases. (B) GC content distribution. The x-axis represents the GC content (%), which was counted over sliding windows, with a window size of 500 bp and a step size of 250 bp. The y-axis represents the ratio of the number of windows corresponding to the GC content to the total number of windows. (C) Distribution of GC content along the sequencing depth. The x-axis and y-axis represent the GC content and sequencing depth, respectively, both of which were counted in 5-kb windows.


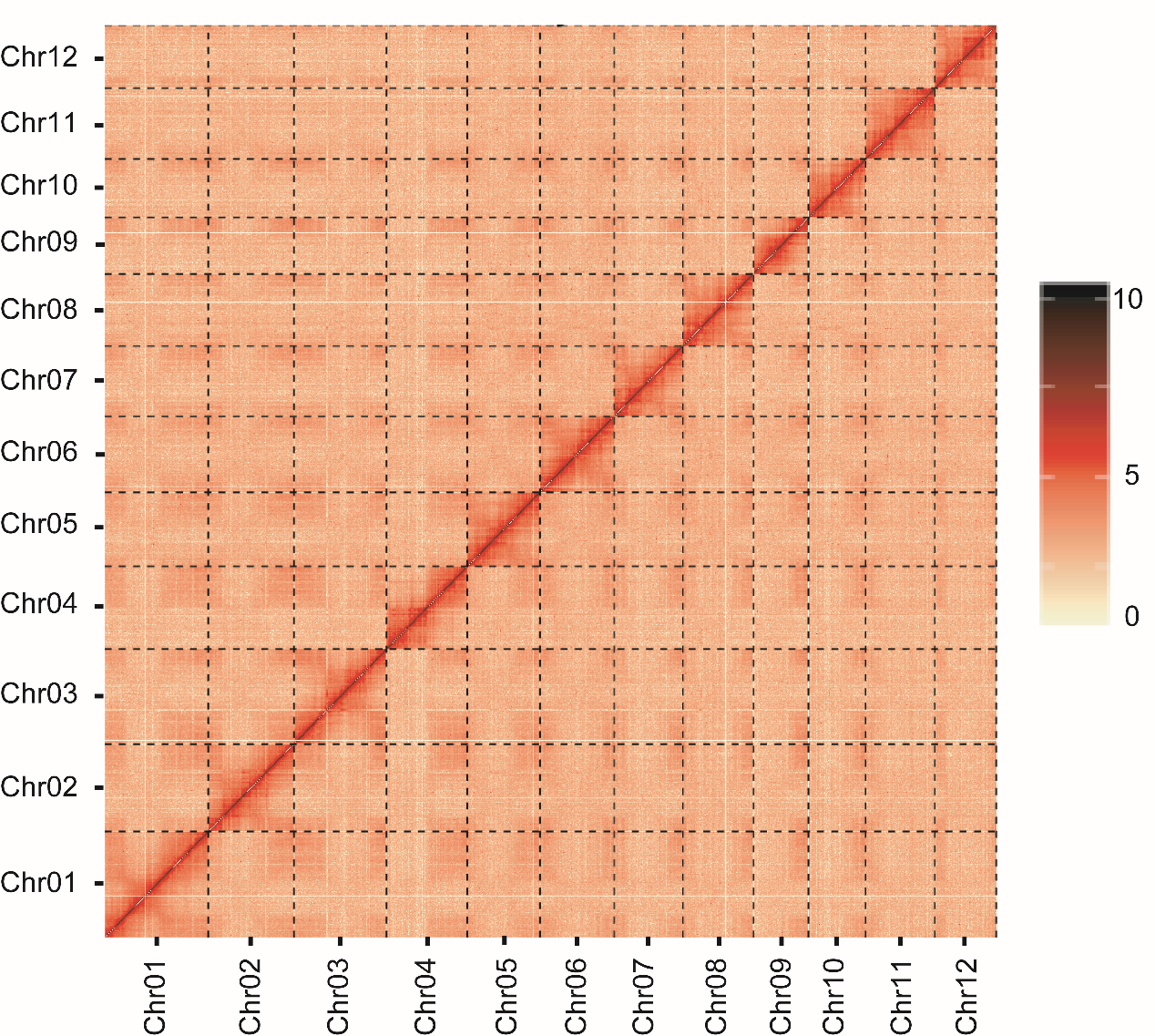


**Figure S7** Hi-C heatmap displaying genome-wide all-by-all interactions among the 12 chromosomes of LM8. The heatmap coordinates represent the chromosomes, and each point in the map represents the log value of the interaction intensity of the corresponding genome bin pair. the interaction intensity increases from red to black (right panel).


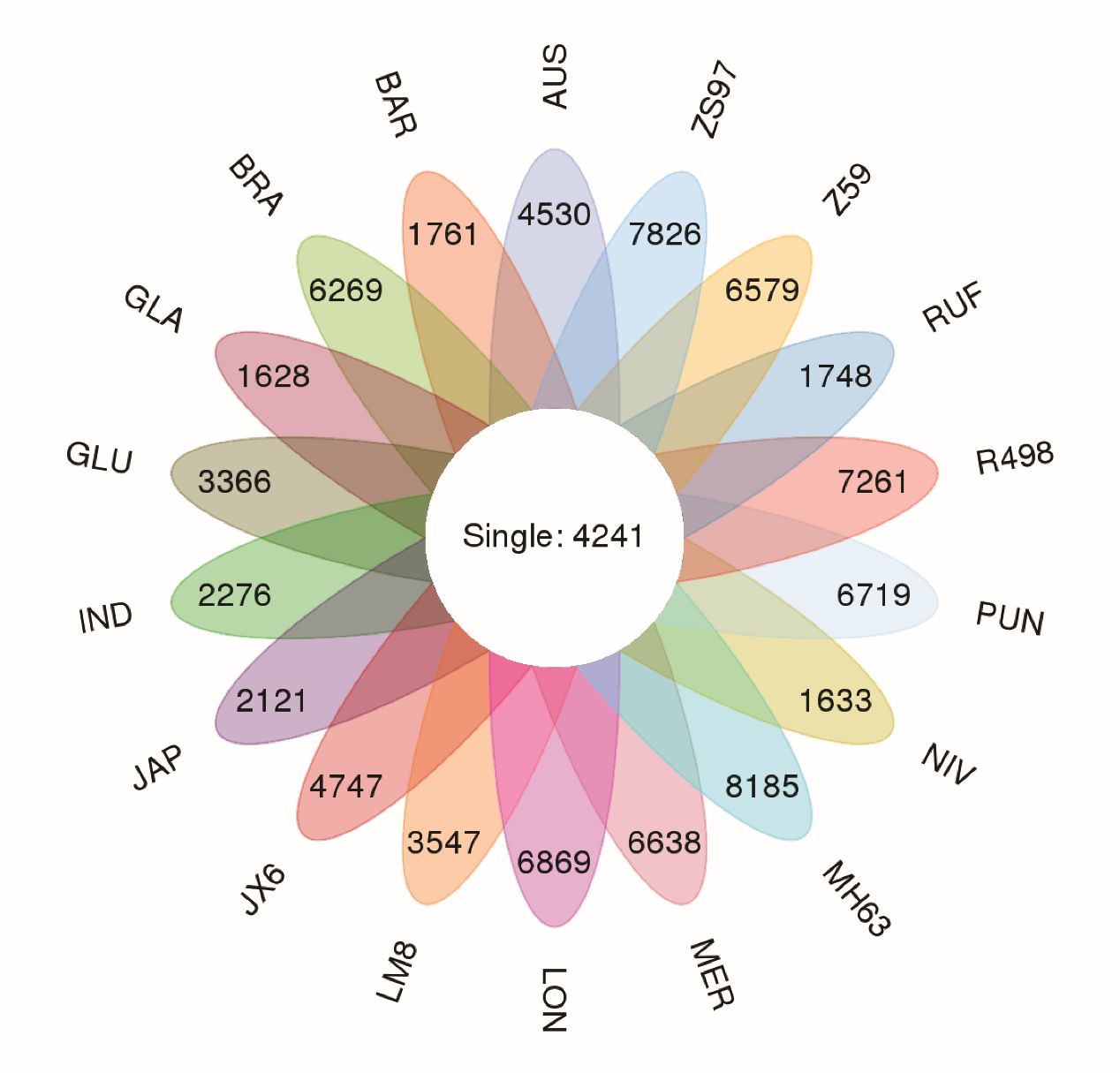


**Figure S8** Petal diagram of single-copy genes and species-specific genes. The number in the middle represents the number of single-copy genes, and the numbers on the petals represent the sum of specific and unclustered genes. The abbreviations are the same as those in Figure S6.


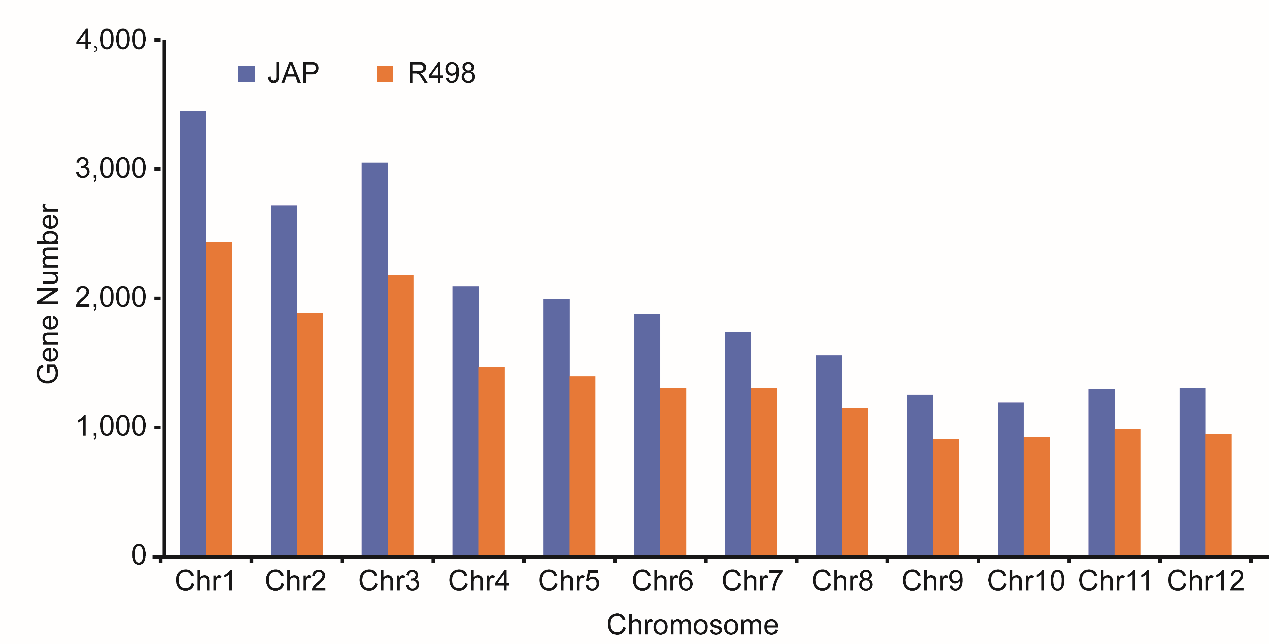


**Figure S9** Statistics of collinear genes between LM8 and two cultivated rice (JAP and R498) genomes. The horizontal axis represents the 12 chromosomes of the genome. The vertical axis represents the number of collinear genes on the same chromosome.
