# Supplementary material for: High-Quality Genomes and High-Density Genetic Map Facilitate the Identification of Genes From a Weedy Rice
Source: Front Plant Sci. 2021 Nov 19;12:775051. doi: 10.3389/fpls.2021.775051 (PMC8639688; doi:10.3389/fpls.2021.775051)
Supplement: Supplementary file 2 [file Data_Sheet_2.docx]

**Table S1** Phenotypic characteristics of LM8 and Shen 08S. * P < 0.05. ** P < 0.01.

|  | GL (mm) | GW (mm) | LWR | GT (mm) | TGW | PH (cm) | PL (cm) | TN | PB | SB | FLL (cm) | FLW (cm) |
| --- | --- | --- | --- | --- | --- | --- | --- | --- | --- | --- | --- | --- |
| LM8 | 5.81 | 2.04 | 2.86 | 1.76 | 10.32 | 141 | 30.8 | 11 | 12 | 36 | 37.5 | 1.1 |
| Shen08s | 8.47 | 2.73 | 3.12 | 2.11 | 22.13 | 65.4 | 25.3 | 13 | 13 | 26 | 49.9 | 1.8 |
| P_Value | ** | ** | ** | ** | ** | ** | ** | * | * | * | ** | ** |
| * *P*<0.05. ** *P*<0.01 | | |  |  |  |  |  |  |  |  |  |  |

**Table S2** Mapping intervals containing important QTLs. PVE, phenotypic variance explained.

| Trait | Chromosome | Geneetic Interval (cM) | Physical Interval (Mb) | Position (Mb) | LOD | Additive | PVE |
| --- | --- | --- | --- | --- | --- | --- | --- |
| GL | LG03 | 788.3-789.4 | 18.81-18.87 | 788.51 | 12.664495 | 0.4535 | 19.8757 |
| GW | LG01 | 413.4-426.2 | 9.33-9.34 | 422.11 | 5.954397 | 0.0758 | 6.204 |
| GW | LG08 | 184.3-197.7 | 28.76-29.22 | 184.61 | 9.680782 | 0.106 | 12.6482 |
| GW | LG11 | 243.3-269.6 | 5.52-6.69 | 252.01 | 6.703583 | -0.0924 | 12.7571 |
| GT | LG01 | 655-661.3 | 19.04-19.1 | 657.81 | 4.102063 | 0.0409 | 10.1391 |
| GT | LG01 | 1060.4-1078.5 | 19.65-19.67 | 1064.41 | 4.184582 | -0.1041 | 0.0001 |
| GT | LG03 | 785.1-789.4 | 18.81-18.93 | 788.51 | 4.197611 | 0.0284 | 8.3976 |
| GT | LG07 | 413.8-427.3 | 22.32-22.52 | 419.91 | 4.612378 | 0.0577 | 6.899 |
| GT | LG09 | 762.9-780 | 19.17-19.36 | 764.91 | 5.224756 | -0.0419 | 0.9647 |
| GT | LG11 | 34.4-37.5 | 3.48-3.48 | 36.11 | 6.123779 | -0.0563 | 17.3557 |
| LWR | LG03 | 764.3-766.2 | 19.34-19.35 | 765.51 | 10.015201 | 0.2307 | 0 |
| TGW | LG03 | 782.9-786.6 | 22.87-22.91 | 785.61 | 22.749186 | 2.7575 | 34.7431 |
| TGW | LG03 | 787.6-788.2 | 18.85-18.87 | 787.91 | 23.897937 | 2.7714 | 36.487 |
| TGW | LG11 | 244.6-253 | 5.52-5.53 | 251.01 | 11.589577 | -2.5234 | 18.845 |
| PL | LG02 | 204.9-217.6 | 25.71-25.73 | 210.11 | 4.568947 | 2.455 | 30.927 |
| PL | LG09 | 391.7-400.8 | 15.54-15.55 | 395.61 | 3.648208 | -1.4294 | 10.6295 |
| PH | LG01 | 254.1-278.9 | 0.56-0.62 | 264.21 | 3.986971 | -8.0743 | 9.7818 |
| PH | LG03 | 56.6-72.8 | 38.22-38.29 | 61.51 | 4.399566 | -7.2696 | 3.7284 |
| PB | LG01 | 1233.9-1248.4 | 21.97-22.42 | 1233.91 | 4.907709 | 0.8325 | 8.0253 |
| PB | LG04 | 889.5-908.1 | 1.75-1.94 | 898.81 | 4.241042 | 0.5769 | 0.1237 |
| PB | LG06 | 388.1-406.7 | 6.54-6.85 | 399.91 | 5.520087 | -0.8699 | 13.4689 |
| SB | LG03 | 39.1-42.7 | 37.84-37.93 | 41.61 | 3.986971 | -4.1535 | 0.6317 |
| SB | LG08 | 11.4-17.3 | 28.81-28.81 | 13.31 | 7.526602 | 10.4014 | 21.0274 |
| TN | LG04 | 149.3-152.8 | 29.58-29.71 | 151.31 | 3.59392 | 1.2062 | 2.6483 |
| TN | LG04 | 490.9-496.6 | 26.92-26.94 | 493.91 | 5.700326 | -3.011 | 4.2384 |
| TN | LG08 | 13.3-36.3 | 28.81-28.97 | 27.01 | 3.624321 | 1.0088 | 8.3151 |
| TN | LG12 | 389.7-398.9 | 3.86-3.93 | 394.41 | 3.596091 | -1.1681 | 7.0452 |
| FLL | LG03 | 4.7-48.2 | 37.7-37.93 | 37.81 | 3.252986 | -3.6113 | 5.9883 |
| FLL | LG08 | 447.6-447.6 | 6.25-8.07 | 458.21 | 3.140065 | 2.5263 | 1.9074 |
| FLL | LG11 | 793.7-797.7 | 22.58-22.58 | 796.31 | 3.880565 | 0.9566 | 1.4456 |
| FLW | LG01 | 1218.7-1235 | 18.97-18.97 | 1223.51 | 7.02063 | 0.1695 | 5.1806 |
| FLW | LG11 | 33.6-38 | 3.48-3.48 | 36.11 | 6.636265 | -0.1494 | 18.0403 |
| PVE, phenotypic variance axplained | | | |  |  |  |  |

**Table S3** Correlation analyses between phenotypic traits.

|  | TGW | GL | GW | LWR | GT | TN | PL | PB | SB | PH | FLL | FLW |
| --- | --- | --- | --- | --- | --- | --- | --- | --- | --- | --- | --- | --- |
| TGW | 1 | 0.829 | 0.368 | 0.674 | 0.673 | 0.136 | 0.348 | 0.381 | 0.202 | 0.249 | 0.205 | 0.44 |
| GL | 0.829 | 1 | 0.224 | 0.908 | 0.528 | 0.095 | 0.248 | 0.224 | 0.029 | 0.11 | 0.113 | 0.283 |
| GW | 0.368 | 0.224 | 1 | -0.198 | 0.322 | 0.051 | 0.137 | 0.341 | 0.264 | -1E-04 | 0.123 | 0.457 |
| LWR | 0.674 | 0.908 | -0.198 | 1 | 0.395 | 0.073 | 0.19 | 0.072 | -0.091 | 0.104 | 0.06 | 0.091 |
| GT | 0.673 | 0.528 | 0.322 | 0.395 | 1 | -0.003 | 0.215 | 0.35 | 0.144 | 0.075 | 0.216 | 0.411 |
| TN | 0.136 | 0.095 | 0.051 | 0.073 | -0.003 | 1 | 0.287 | 0.294 | 0.416 | 0.351 | 0.213 | 0.211 |
| PL | 0.348 | 0.248 | 0.137 | 0.19 | 0.215 | 0.287 | 1 | 0.539 | 0.526 | 0.599 | 0.322 | 0.195 |
| PB | 0.381 | 0.224 | 0.341 | 0.072 | 0.35 | 0.294 | 0.539 | 1 | 0.587 | 0.439 | 0.321 | 0.498 |
| SB | 0.202 | 0.029 | 0.264 | -0.091 | 0.144 | 0.416 | 0.526 | 0.587 | 1 | 0.396 | 0.313 | 0.444 |
| PH | 0.249 | 0.11 | -1E-04 | 0.104 | 0.075 | 0.351 | 0.599 | 0.439 | 0.396 | 1 | 0.199 | 0.094 |
| FLL | 0.205 | 0.113 | 0.123 | 0.06 | 0.216 | 0.213 | 0.322 | 0.321 | 0.313 | 0.199 | 1 | 0.204 |
| FLW | 0.44 | 0.283 | 0.457 | 0.091 | 0.411 | 0.211 | 0.195 | 0.498 | 0.444 | 0.094 | 0.204 | 1 |

**Table S4** Location and annotation of the seven genes in the candidate QTL interval.

| Chromosome | Gene | RAP_Locus | Gene annotation |
| --- | --- | --- | --- |
| lg03 | ORUFILM03g000090 | - | - |
| lg03 | ORUFILM03g000091 | Os03g0428200 | terpene synthase, N-terminal domain containing protein, expressed |
| lg03 | ORUFILM03g000092 | - | - |
| lg03 | ORUFILM03g000093 | - | - |
| lg03 | ORUFILM03g000094 | - | - |
| lg03 | ORUFILM03g000095 | Os03g0427900 | U-box domain-containing protein, putative, expressed |
| lg03 | ORUFILM03g000096 | Os03g0427300 | glutelin, putative, expressed |

**Table S5** Summary of the sequencing of the LM8 genome.

| Stat Type | Depth | Date Size |
| --- | --- | --- |
| Illumina hiseq X10 | 50X | 20G |
| Nanopore sequencing | 100X | 40G |
| BioNano sequencing | 200X | 4Gb |
| HiC sequencing | 201X | 80G |
| Whole transcriptome sequencing | 20X | 80G |

**Table S6** Statistics of the LM8 genome assembly.

| Stat Type | Nanopore Length (bp) | Nanopore Number | BioNano Length (bp) | BioNano Number | HiC  Length (bp) | HiC Number |
| --- | --- | --- | --- | --- | --- | --- |
| N50 | 17863247 | 8 | 24066034 | 7 | 30527306 | 6 |
| N60 | 14283707 | 11 | 22376122 | 9 | 29589233 | 7 |
| N70 | 13655952 | 13 | 17863247 | 11 | 29120070 | 8 |
| N80 | 12039701 | 16 | 17122943 | 13 | 28930374 | 9 |
| N90 | 9797334 | 20 | 13655952 | 15 | 24066034 | 11 |
| Longest | 31271747 | 1 | 31271747 | 1 | 43711559 | 1 |
| **Total** | **375300810** | **35** | **375770508** | **30** | **375771908** | **16** |

**Table S7** Assessment of the LM8 genome assembly.

| Genome assembly assessment | Assembly Number | Assembly Percent(%) | Annotion Number | Annotion Percent(%) |
| --- | --- | --- | --- | --- |
| Complete BUSCOs (C) | 1349 | 98.1 | 1347 | 97.9 |
| Complete and single-copy BUSCOs (S) | 1321 | 96.1 | 1326 | 96.4 |
| Complete and duplicated BUSCOs (D) | 28 | 2 | 1326 | 1.5 |
| Fragmented BUSCOs (F) | 5 | 0.4 | 11 | 0.8 |
| Missing BUSCOs (M) | 21 | 1.5 | 17 | 1.3 |
| **Total BUSCO groups searched** | **1,375** | **-** | **1,375** | **100** |

**Table S8** Statistics of the length of the 12 chromosomes of LM8.

| Group ID | Length (bp) | Scaffold Number |
| --- | --- | --- |
| lg01 | 43711559 | 3 |
| lg02 | 36032174 | 2 |
| lg03 | 38965438 | 3 |
| lg04 | 34022783 | 2 |
| lg05 | 30527306 | 2 |
| lg06 | 31271747 | 1 |
| lg07 | 28930374 | 3 |
| lg08 | 29589233 | 3 |
| lg09 | 23102722 | 1 |
| lg10 | 24066034 | 1 |
| lg11 | 29120070 | 2 |
| lg12 | 25875407 | 3 |
| **Total** | **375214847** | **26** |

**Table S9** Statistics of the repeat annotation.

| **Class** | **Order** | **Super family** | **Number of elements** | **Length of sequence(bp)** | **Percentage of sequence(%)** |
| --- | --- | --- | --- | --- | --- |
| **Class I, Retrotransposon** |  |  | **123178** | **100966255** | **26.87** |
|  | LTR |  | 105780 | 95001588 | 25.28 |
|  |  | Copia | 17492 | 11228976 | 2.99 |
|  |  | Gypsy | 65605 | 73722592 | 19.62 |
|  |  | Caulimovirus | 338 | 461764 | 0.12 |
|  |  | Unknown | 21590 | 9426911 | 2.51 |
|  |  | Other | 755 | 161345 | 0.04 |
|  | LINE |  | 13787 | 5441339 | 1.45 |
|  |  | L1 | 13373 | 5362484 | 1.43 |
|  |  | Other | 414 | 78855 | 0.02 |
|  | SINE |  | 3611 | 523328 | 0.14 |
|  |  | Other | 3611 | 523328 | 0.14 |
| **Class II, DNA Transposon** |  |  | **326093** | **78335650** | **20.85** |
|  | DNA |  | 255999 | 61365127 | 16.33 |
|  |  | PIF-Harbinger | 54668 | 10717738 | 2.85 |
|  |  | MULE-MuDR | 60770 | 16344126 | 4.35 |
|  |  | CMC-EnSpm | 33349 | 15247200 | 4.06 |
|  |  | hAT-Tip100 | 12442 | 1762255 | 0.47 |
|  |  | TcMar-Stowaway | 53890 | 8199186 | 2.18 |
|  |  | hAT-Ac | 9721 | 2648312 | 0.7 |
|  |  | hAT-Tag1 | 2475 | 456212 | 0.12 |
|  |  | Unknown | 26050 | 5413150 | 1.44 |
|  |  | Other | 2634 | 576948 | 0.15 |
|  | MITE |  | 17682 | 3767530 | 1 |
|  |  | Unknown | 17682 | 3767530 | 1 |
|  | RC |  | 52412 | 13202993 | 3.51 |
|  |  | Helitron | 52367 | 13201088 | 3.51 |
|  |  | Other | 45 | 1905 | 0 |
| **Total TEs** |  |  | **449271** | **179301905** | **47.72** |
| Unknown |  |  | 9569 | 3021913 | 0.8 |
| Simple repeats |  |  | 2022 | 322739 | 0.09 |
| Other |  |  | 2259 | 1158073 | 0.31 |
| Low complexity |  |  | 18 | 2111 | 0 |
| **Total Repeats** |  |  | **463139** | **183806741** | **48.91** |

**Table S10** Statistics of the functional annotation.

| Type | Number | Percent (%) |
| --- | --- | --- |
| Swissprot | 23,531 | 64.36 |
| KEGG | 9,546 | 26.11 |
| KOG | 15,266 | 41.75 |
| GO | 14,738 | 40.31 |
| NR | 34,738 | 95.01 |
| Annotated | 34,773 | 95.11 |
| Unannotion | 1,788 | 4.89 |
| **Total** | **36,561** | **100** |

**Table S11** Statistics of the non-coding RNA (ncRNA) annotation.

| Type | | Copy | Average Length(bp) | Total Length(bp) | Content |
| --- | --- | --- | --- | --- | --- |
| rRNA | rRNA | 81 | 564.85 | 45753 | 0.008702 |
|  | 18S | 9 | 1703.22 | 15329 | 0.002915 |
|  | 28S | 6 | 3779.00 | 22674 | 0.004312 |
|  | 5.8S | 5 | 155.40 | 777 | 0.000148 |
|  | 5S | 61 | 114.31 | 6973 | 0.001326 |
| snRNA | snRNA | 772 | 112.88 | 87147 | 0.016574 |
|  | CD-box | 626 | 106.39 | 66602 | 0.012667 |
|  | HACA-box | 63 | 130.79 | 8240 | 0.001567 |
|  | splicing | 83 | 148.25 | 12305 | 0.00234 |
| miRNA | | 2551 | 187.20 | 477538 | 0.090823 |

**Table S12** Statistics of gene families in the LM8 genome.

| Type | Single | Multi | Unique | Other | Unclustered |
| --- | --- | --- | --- | --- | --- |
| AUS | 4241 | 8062 | 217 | 19307 | 4313 |
| BAR | 4241 | 9384 | 33 | 25095 | 1728 |
| BRA | 4241 | 8122 | 531 | 11188 | 5738 |
| GLA | 4241 | 8904 | 14 | 23060 | 1614 |
| GLU | 4241 | 9393 | 182 | 24158 | 3184 |
| IND | 4241 | 9444 | 18 | 21516 | 2258 |
| JAP | 4241 | 9218 | 32 | 23176 | 2089 |
| JX6 | 4241 | 9665 | 58 | 20599 | 4689 |
| LM8 | 4241 | 10093 | 672 | 19798 | 2875 |
| LON | 4241 | 9299 | 214 | 11277 | 6655 |
| MER | 4241 | 9410 | 420 | 21687 | 6218 |
| MH63 | 4241 | 9483 | 824 | 14974 | 7361 |
| NIV | 4241 | 9029 | 8 | 21410 | 1625 |
| PUN | 4241 | 8872 | 457 | 15259 | 6262 |
| R498 | 4241 | 9848 | 737 | 16989 | 6524 |
| RUF | 4241 | 9009 | 12 | 22067 | 1736 |
| Z59 | 4241 | 9443 | 65 | 19080 | 6514 |
| ZS97 | 4241 | 9325 | 651 | 13515 | 7175 |

**Table S13** Statistics of the genome assembly of the 18 *Oryza* genomes.

| Abbreviation | Genome type | Assmbly length(bp) | Contig count | Contig N50(bp) | Scaffold count | Scaffold N50(bp) |
| --- | --- | --- | --- | --- | --- | --- |
| MH63 | AA | 359,918,891 | 181 | 3,097,358 | NA | NA |
| ZS97 | AA | 346,854,256 | 237 | 2,339,070 | NA | NA |
| R498 | AA | 390,983,850 | 17 | 25,582,588 | 14 | 31,778,392 |
| JX6 | AA | 410,179,672 | 218 | 5,825,388 | 68 | 21,140,215 |
| Z59 | AA | 397,537,926 | 291 | 2,665,012 | 95 | 13,194,956 |
| LM8 | AA | 375,300,810 | 35 | 17,863,247 | 30 | 24,066,034 |
| IND | AA | 374,545,499 | 35416 | 27,122 | NA | NA |
| JAP | AA | 373,245,519 | 242 | 7,711,345 | NA | NA |
| GLA | AA | 285,037,524 | 21269 | 24,838 | NA | NA |
| AUS | AA | 362,279,097 | 912 | 946,210 | 912 | 946,210 |
| BAR | AA | 308,272,304 | 25427 | 18,926 | 3001 | 443,744 |
| GLU | AA | 372,860,283 | 17912 | 31,921 | 3157 | 451,867 |
| MER | AA | 335,668,232 | 62778 | 9,149 | 18305 | 238,568 |
| RUF | AA | 339,177,042 | 68481 | 34,232 | 49224 | 137,860 |
| NIV | AA | 337,950,324 | 16484 | 37,688 | 2430 | 295,425 |
| LON | AA | 362,063,720 | 26,322 | 39,115 | 11,745 | 363,864 |
| PUN | BB | 393,816,603 | 16598 | 43,035 | 1641 | 1,310,149 |
| BRA | FF | 250,927,218 | 19463 | 21,984 | NA | NA |

**Table S14** Statistics of the expanded and contracted gene families in the 18 *Oryza* genomes.

| Species | Gene  Families | Expansions Families | Expansions Genes | Contractions Families |
| --- | --- | --- | --- | --- |
| AUS | 25804 | 3083 | 6023 | 10449 |
| IND | 27334 | 3993 | 7885 | 4564 |
| NIV | 27868 | 3317 | 6820 | 4030 |
| MH63 | 31958 | 4454 | 17785 | 3995 |
| ZS97 | 30284 | 4271 | 17298 | 5669 |
| R498 | 22921 | 3956 | 8894 | 16410 |
| JAP | 29168 | 3612 | 7499 | 4041 |
| RUF | 28536 | 3305 | 6793 | 4673 |
| LM8 | 25430 | 4086 | 9374 | 7307 |
| JX6 | 26618 | 3792 | 7945 | 3015 |
| Z59 | 25256 | 3705 | 7573 | 4377 |
| BAR | 30843 | 3746 | 7910 | 2435 |
| GLA | 29045 | 3441 | 7174 | 4233 |
| GLU | 29280 | 3858 | 8694 | 7326 |
| LON | 17747 | 0 | 0 | 38635 |
| MER | 27066 | 315 | 407 | 29680 |
| PUN | 21277 | 1158 | 1669 | 36693 |
| BRA | 18024 | 788 | 1028 | 40488 |

**Table S15 Enriched GO terms of detected in QTL.**

| Trait | Chromosome | Gene | GO enrichment |
| --- | --- | --- | --- |
| TWG | lg03 | ORUFILM03g004239 | transmembrane transporter activity |
| TWG | lg03 | ORUFILM03g004244 | potassium ion transmembrane transporter activity |
| GW | lg08 | ORUFILM08g002572 | proteolysis |
| GW | lg11 | ORUFILM11g001610 | ADP binding |
| GW | lg11 | ORUFILM11g001611 | ADP binding |
| GW | lg11 | ORUFILM11g001612 | ADP binding |
| GW | lg11 | ORUFILM11g001613 | ADP binding |
| GW | lg11 | ORUFILM11g001616 | ADP binding |
| GW | lg11 | ORUFILM11g001617 | ADP binding |
| GW | lg11 | ORUFILM11g001619 | ADP binding |
| GW | lg11 | ORUFILM11g001621 | ADP binding |
| GW | lg11 | ORUFILM11g001623 | ADP binding |
| GW | lg11 | ORUFILM11g001624 | ADP binding |
| GW | lg11 | ORUFILM11g001629 | ADP binding |
| GW | lg11 | ORUFILM11g001630 | ADP binding |
| GW | lg11 | ORUFILM11g001632 | ADP binding |
| GW | lg11 | ORUFILM11g001638 | ADP binding |
| GW | lg11 | ORUFILM11g001639 | ADP binding |
| GW | lg11 | ORUFILM11g001672 | magnesium ion binding |
| GW | lg11 | ORUFILM11g001687 | ADP binding |
| GW | lg11 | ORUFILM11g001693 | ADP binding |
| GW | lg11 | ORUFILM11g001695 | ADP binding |
| GT | lg01 | ORUFILM01g001943 | ADP binding |
| GT | lg07 | ORUFILM07g001970 | carbohydrate binding |
| GT | lg07 | ORUFILM07g001973 | carbohydrate binding |
| GT | lg07 | ORUFILM07g001990 | copper ion binding |
| GT | lg09 | ORUFILM09g001483 | coenzyme binding |
| GT | lg09 | ORUFILM09g001487 | catalytic activity |
| TN | lg04 | ORUFILM04g001130 | pyridoxal phosphate binding |
| TN | lg04 | ORUFILM04g001131 | pyridoxal phosphate binding |
| TN | lg04 | ORUFILM04g001134 | potassium ion transmembrane transporter activity |
| TN | lg08 | ORUFILM08g002572 | proteolysis |
| PB | lg01 | ORUFILM01g002170 | transmembrane transporter activity |
| PB | lg01 | ORUFILM01g002171 | transmembrane transporter activity |
| PB | lg01 | ORUFILM01g002209 | proteolysis |
| PB | lg01 | ORUFILM01g002210 | proteolysis |
| PB | lg04 | ORUFILM04g000378 | flavin adenine dinucleotide binding |
| Trait | Chromosome | Gene | GO enrichment |
| PB | lg06 | ORUFILM06g000875 | carbohydrate binding |
| SB | lg03 | ORUFILM03g002495 | transferase activity, transferring hexosyl groups |
| SB | lg03 | ORUFILM03g002497 | polysaccharide binding |
| SB | lg03 | ORUFILM03g002499 | catalytic activity |
| PH | lg01 | ORUFILM01g000077 | transferase activity, transferring acyl groups other than amino-acyl groups |
| PH | lg03 | ORUFILM03g002433 | iron ion binding |
| PH | lg03 | ORUFILM03g002441 | ADP binding |
| PH | lg03 | ORUFILM03g002445 | acid phosphatase activity |
| PH | lg03 | ORUFILM03g002446 | catalytic activity |
| FLL | lg03 | ORUFILM03g002495 | transferase activity, transferring hexosyl groups |
| FLL | lg03 | ORUFILM03g002497 | polysaccharide binding |
| FLL | lg03 | ORUFILM03g002499 | catalytic activity |
| FLL | lg03 | ORUFILM03g002518 | ammonium transmembrane transporter activity |
| FLL | lg03 | ORUFILM03g002519 | recognition of pollen |
| FLL | lg03 | ORUFILM03g002530 | cellulose synthase (UDP-forming) activity |
| FLL | lg08 | ORUFILM08g000735 | pyridoxal phosphate binding |
| FLL | lg08 | ORUFILM08g000736 | potassium ion transmembrane transporter activity |
| FLL | lg08 | ORUFILM08g000795 | ADP binding |
| FLL | lg08 | ORUFILM08g000800 | carbohydrate metabolic process |
| FLL | lg08 | ORUFILM08g000803 | catalytic activity |

**Table S16 Genotype analysis of *ORUFILM03g000095* in 3K database**

| Haplotypes | Sample List | SNPs | Average Grain Length | 17885140 | 17885257 | 17885455 | 17885514 | 17886626 | 17886650 | 17887662 | 17888918 | 17890385 | 17891598 | 17891706 |
| --- | --- | --- | --- | --- | --- | --- | --- | --- | --- | --- | --- | --- | --- | --- |
| LM8 | 1 | CTCTCCTACGG | 5.81 | C | T | C | T | C | C | T | A | C | G | G |
| Hap2 | 1351 | CTCTCCTGTAG | 8.739 | **C** | **T** | **C** | **T** | **C** | **C** | **T** | **G** | **T** | **A** | **G** |
| Hap3 | 658 | CCCATTTGTAG | 8.530 | **C** | **C** | **C** | **A** | **T** | **T** | **T** | **G** | **T** | **A** | **G** |
| Hap4 | 193 | CTCTCCTGCAG | 8.235 | **C** | **T** | **C** | **T** | **C** | **C** | **T** | **G** | **C** | **A** | **G** |
| Hap5 | 151 | CTCTCCTGTGG | 8.730 | **C** | **T** | **C** | **T** | **C** | **C** | **T** | **G** | **T** | **G** | **G** |
| Hap6 | 129 | TCCTCCAGTAG | 8.136 | **T** | **C** | **C** | **T** | **C** | **C** | **A** | **G** | **T** | **A** | **G** |
| Hap7 | 112 | CCCTCCTGTAG | 8.681 | **C** | **C** | **C** | **T** | **C** | **C** | **T** | **G** | **T** | **A** | **G** |
| Hap8 | 47 | CCCTCTTTTAC | 7.950 | **C** | **C** | **C** | **T** | **C** | **T** | **T** | **T** | **T** | **A** | **C** |
| Hap9 | 45 | CTTTCCTGTAG | 8.987 | **C** | **T** | **T** | **T** | **C** | **C** | **T** | **G** | **T** | **A** | **G** |
| Hap10 | 32 | C-CTCCTGTAG | 8.845 | **C** | **-** | **C** | **T** | **C** | **C** | **T** | **G** | **T** | **A** | **G** |
| Hap11 | 19 | CYCTCCTGTAG | 8.700 | **C** | **T** | **C** | **T** | **C** | **C** | **T** | **G** | **T** | **A** | **G** |
| Hap12 | 18 | CYCWYYTGTAG | NA | **C** | **T** | **C** | **T** | **C** | **T** | **T** | **G** | **T** | **A** | **G** |
| Hap13 | 15 | -TCTCCTGTAG | 8.586 | **-** | **T** | **C** | **T** | **C** | **C** | **T** | **G** | **T** | **A** | **G** |
| Hap14 | 13 | CTCTCCTGYAG | 6.200 | **C** | **T** | **C** | **T** | **C** | **C** | **T** | **G** | **Y** | **A** | **G** |
